# Amitriptyline induces mitophagy that precedes apoptosis in human HepG2 cells

**DOI:** 10.18632/genesandcancer.114

**Published:** 2016-07

**Authors:** Marina Villanueva-Paz, Mario D. Cordero, Ana Delgado Pavón, Beatriz Castejón Vega, David Cotán, Mario De la Mata, Manuel Oropesa-Ávila, Elizabet Alcocer-Gomez, Isabel de Lavera, Juan Garrido-Maraver, José Carrascosa, Ana Paula Zaderenko, Jordi Muntané, Manuel de Miguel, José Antonio Sánchez-Alcázar

**Affiliations:** ^1^ Centro Andaluz de Biología de Desarrollo (CABD), Universidad Pablo de Olavide/CSIC/, Sevilla, Spain; ^2^ Facultad de Odontología, Universidad de Sevilla, Sevilla, Spain; ^3^ Sistemas Físicos, Químicos y Naturales-Universidad Pablo de Olavide, Sevilla, Spain; ^4^ Departmento de Cirugía General y Aparato Digestivo, Hospital Universitario Virgen del Rocío/Instituto de Biomedicina de Sevilla (IBiS)/CSIC/Universidad de Sevilla, Sevilla, Spain; ^5^ Departamento de Citología e Histología Normal y Patológica, Facultad de Medicina, Universidad de Sevilla, Sevilla, Spain; ^6^ Centro de Investigación Biomédica en Red de Enfermedades Hepáticas y Digestivas (CIBEREHD), Madrid, Spain; ^7^ Centro de Investigación Biomédica en Red de Enfermedades Raras (CIBERER), ISCIII, Madrid, Spain

**Keywords:** mitophagy, apoptosis, amitriptyline, oxidative stress, HepG2 cells

## Abstract

Systemic treatments for hepatocellular carcinoma (HCC) have been largely unsuccessful. This study investigated the antitumoral activity of Amitriptyline, a tricyclic antidepressant, in hepatoma cells. Amitriptyline-induced toxicity involved early mitophagy activation that subsequently switched to apoptosis. Amitriptyline induced mitochondria dysfunction and oxidative stress in HepG2 cells. Amitriptyline specifically inhibited mitochondrial complex III activity that is associated with decreased mitochondrial membrane potential (∆Ψm) and increased reactive oxygen species (ROS) production. Transmission electron microscopy (TEM) studies revealed structurally abnormal mitochondria that were engulfed by double-membrane structures resembling autophagosomes. Consistent with mitophagy activation, fluorescence microscopy analysis showed mitochondrial Parkin recruitment and colocalization of mitochondria with autophagosome protein markers.

Pharmacological or genetic inhibition of autophagy exacerbated the deleterious effects of Amitriptyline on hepatoma cells and led to increased apoptosis. These results suggest that mitophagy acts as an initial adaptive mechanism of cell survival. However persistent mitochondrial damage induced extensive and lethal mitophagy, autophagy stress and autophagolysome permeabilization leading eventually to cell death by apoptosis. Amitriptyline also induced cell death in hepatoma cells lines with mutated p53 and non-sense p53 mutation.

Our results support the hypothesis that Amitriptyline-induced mitochondrial dysfunction can be a useful therapeutic strategy for HCC treatment, especially in tumors showing p53 mutations and/or resistant to genotoxic treatments.

## INTRODUCTION

Liver cancer in men is the fifth most frequently diagnosed cancer worldwide but the second most frequent cause of cancer death [1]. Hepatocellular carcinoma (HCC) accounts for 70-85% of the total liver cancer burden [2]. Different situations, such as the presence of agonistic antibodies or soluble death ligand proteins, prevent induction of death receptor-mediated apoptosis in HCCs [3]. HCCs also display high resistance to cell death mediated by tumor necrosis factor-related apoptosis-inducing ligand (TRAIL) [4], which, together with other apoptosis resistance mechanisms, suggests that new approaches are needed to control HCC tumors and metastasis.

A new anticancer strategy named *oxidative therapy* has been developed by inducing cytotoxic oxystress for cancer treatment [5]. It could be achieved by two methods, inducing the generation of high level of reactive oxygen species (ROS) or inhibiting the antioxidant system in tumor cells [6]. It is well known that ROS and their derivatives, such as hydrogen peroxide (H_2_O_2_) and superoxide anion ${(\text{O}}_{2}^{-})$, induce apoptosis to a wide range of tumor cells *via* caspase activation [7]. Since mitochondria are an important source of reactive oxygen intermediates because they are the major consumers of molecular oxygen, mitochondrial damage induced by using mito-targeted drugs may provoke an increase of oxidative stress and cell death [8].

Amitriptyline is a tricyclic antidepressant commonly prescribed for depression and several neuropathic and inflammatory illnesses such as fibromyalgia, chronic fatigue syndrome, migraine, irritable bowel syndrome, and atypical facial pain [9]. However, several reports have demonstrated that Amitriptyline is cytotoxic by increasing oxidative stress and lipid peroxidation [12–12]. In fact, tricyclic antidepressants have been shown to cause apoptotic cell death in normal human lymphocytes [13], non-Hodkin's lymphoma cells [14], and neurons [15]. In addition, previous works of or group have shown that Amitriptyline could be a good candidate for oxidative therapy because its cytotoxicity has been proved to be more effective than other chemotherapeutic drugs in lung cancer H460 cells [10].

The purpose of the present work was to determine the cytotoxicity activity induced by Amitriptyline using hepatoma cells in order to evaluate its potential use for HCC treatment.

## RESULTS

### Amitriptyline induced cell death in HepG2

**Figure 1:**
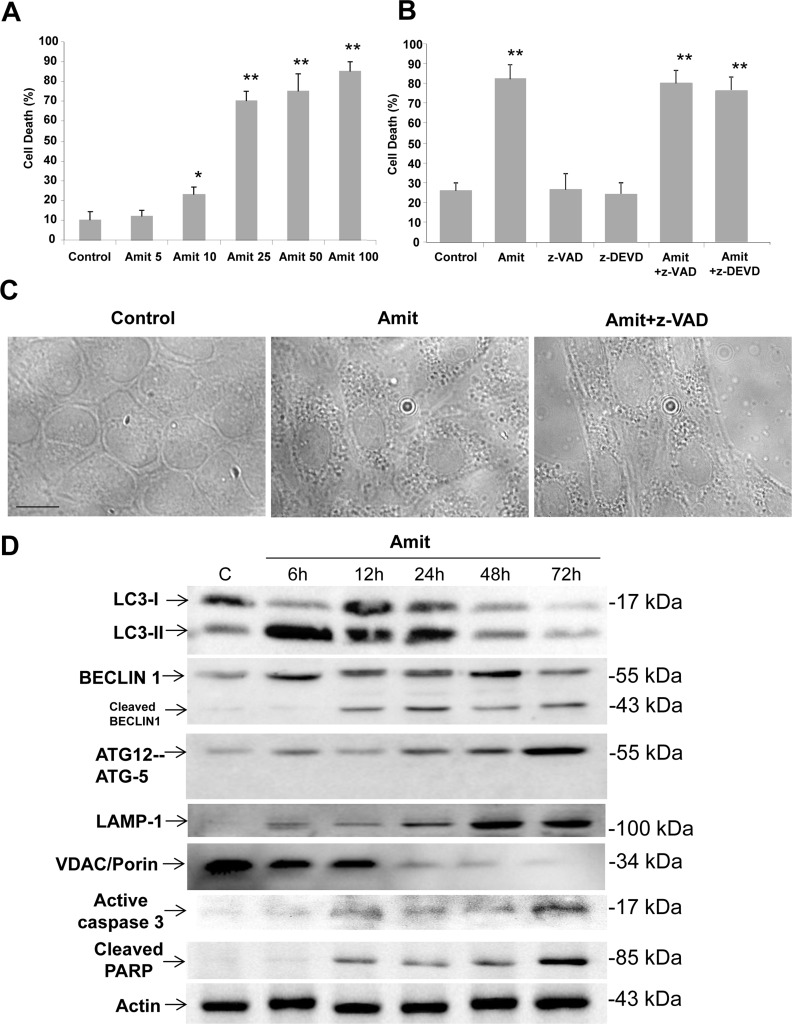
Amitriptyline reduces HepG2 cell viability **A.** Cells were seeded in six-multiwell plates, at a density of 100,000 cells/well. After 24 h of culture, serial concentrations of Amitriptyline (Amit) (0, 5, 10, 25, 50 and 100 μM) were added to the culture medium and cells were further incubated for 24h. Cells were then harvested and viability was analyzed by using the vital dye exclusion assay as described in the Materials and methods section. **B.** Caspase inhibition does not prevent Amitriptyline-induced cell death. HepG2 cells were treated with 50 μM Amitriptyline in the presence of z-VAD (50 μM) or z-DEVD (50 μM) for 24h. Cells were then harvested and viability was analyzed as described in the Materials and methods section. **C.** Phase-contrast light microscopy of HepG2 cells treated with Amitriptyline. Control cells, not exposed to Amitriptyline, showing no vacuolation. Cells exposed to 50 μM Amitriptyline for 6 hours, showing vacuolation. The treatment with 50 μM z-VAD did not prevent Amitriptyline induced vacuolation. **D.** Amitriptyline induces autophagy and apoptosis in HepG2 cells. Expression levels of protein markers of autophagy (LC3, BECLIN 1 and ATG12-ATG5), lysosomes (LAMP-1), mitochondria (VDAC/Porin) and apoptosis (active caspase 3 and cleaved PARP) were examined in HepG2 cells treated with 50 μM Amitriptyline for 6, 12, 24 and 48 hours by Western blotting. Actin was used as loading control.

To assess whether Amitriptyline has cytotoxic activity, HepG2 cells were exposed to increasing concentrations of Amitriptyline (5, 10, 25, 50 and 100 μM) for 24 h and then cell viability was evaluated by trypan blue staining. Microscopic analysis showed that Amitriptyline dose-dependently increased the population of tryplan blue-stained HepG2 cells (Figure 1A). Amitriptyline-induced cell death was not reduced in the presence of the caspases inhibitor z-VAD-fmk or z-DEVD-fmk (Figure 1B). These data suggest that Amitriptyline may induce caspase-independent cell death in HepG2 cells when the apoptotic program is blocked. During these experiments, we observed that Amitriptyline caused profound vacuolization that occurred even before cell death and after administration of z-VAD-fmk, all common features of autophagy activation (Figure 1C).

### Autophagy apoptosis switch by Amitriptyline

To further verify whether early autophagic activation preceding apoptosis was involved in Amitriptyline-induced cell death, we examined both autophagic and apoptotic executive protein expression levels at 6, 12, 24, 48 and 72h after Amitriptyline treatment (Figure 1D). Immunoblotting assays indicated that Amitriptyline treatment induced an early increased in autophagic BECLIN 1, ATG12-ATG-5 and LC3-II protein expression levels (with a peak at 6 h for LC3-II and BECLIN 1) suggesting early autophagy activation. Expression levels of LAMP-1, a lysosomal marker, were also increased. However, expression levels of VDAC/Porin (voltage-dependent anion channel), a mitochondrial marker, decreased gradually after Amitriptyline treatment. After 12 h of treatment there was an increased in caspase 3 activation and cleaved of BECLIN 1 and PARP, a nuclear protein which is typically cleaved during apoptosis. Anti-apoptotic proteins Bcl-x, Survivin and Mcl-1 were also down-regutated after 12-24 hours of Amitriptyline treatment (Supplementary Figure 1A). However, Bcl-2 was slightly increased. Caspase-dependent degradation of BECLIN 1 and anti-apoptotic proteins was confirmed by assessing that protein cleavage was abolished by the concomitant treatment of Amitriptyline with z-VAD (Supplementary Figure 1B and 2).

These results suggest that Amitriptyline induced an early (< 12h) autophagic activation that preceded apoptosis, a process known as autophagy-apoptosis switch [16]. Immunofluorescence analysis also confirmed the presence of cells with both autophagic (discrete LC3 signal) and apoptosis (cytochrome c release and nuclear condensation) activation markers after Amitriptyline treatment (Supplementary Figure 3).

Interestingly, Amitriptyline also induced cell death when apoptosis was blocked by z-VAD suggesting that, in these experimental conditions, Amitriptyline killed HepG2 cells by extensive autophagy (accumulation of autophagic markers) that eventually led to necrosis.

### Cathepsin B undergoes relocalization to the cytosol following Amitriptyline treatment

**Figure 2:**
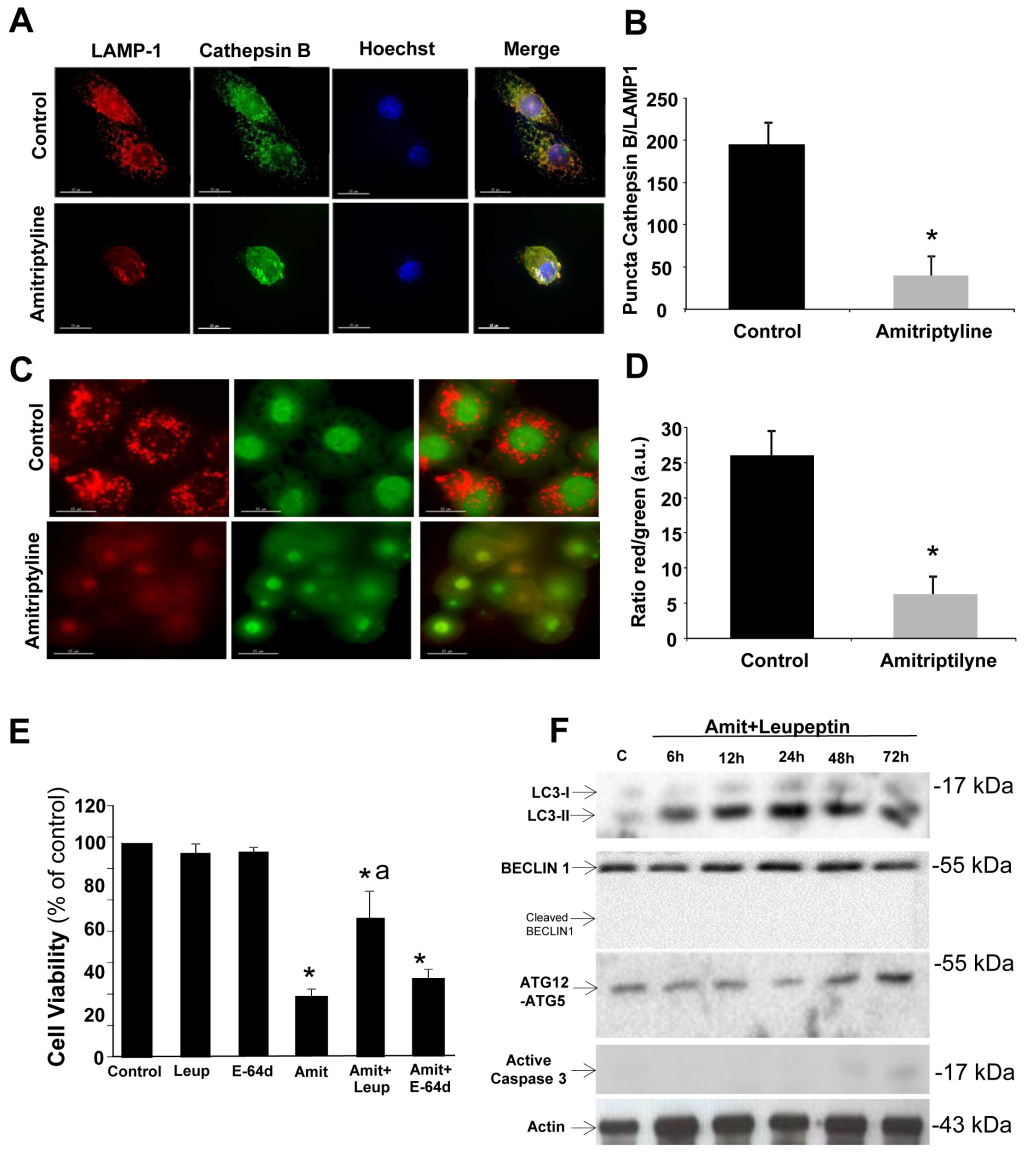
Lysosomal permeabilization detection by immunofluorescence **A.** HepG2 cells were treated with 50 μM Amitriptyline for 12 hours, fixed and stained for immunofluorescence detection of cathepsin B (green) and LAMP-1 (red). Note that after Amitriptyline treatment, cathepsin B diffuses throughout the cytosol. **B.** Quantification of colocalization puncta of cathepsin B and LAMP-1 by immunofluorescence microscopy. **C.** HepG2 cells were cultured for 12 h with 50μM Amitriptyline and stained for 15 min with 10 μg/ ml acridine orange. Note the loss of red staining puncta after Amitriptyline treatment. **D.** Quantification of the ratio between the red and green signal of acridine orange was performed by immunofluorescence microscopy using the Image J software. *p < 0.01 between control and amitriptyline-treated cells. **E.** Cells were seeded in six-multiwell plates, at a density of 100,000 cells/well. After 24 h culture, 50 μM Amitriptyline (Amit), were added to the culture medium in the presence or absence of 100 μM leupeptin (Leup) or 100 μM E-64d and cells were further incubated for 24h. Cells were then harvested and viability was analyzed by using the vital dye exclusion assay as described in the Materials and methods section. **F.** Expression levels of protein markers of autophagy (LC3, BECLIN 1 and ATG12-ATG5) and apoptosis (active caspase 3) were examined in HepG2 cells treated with 50 μM Amitriptyline (Amit) and 100 μM leupeptin for 6, 12, 24 and 48 hours by Western blotting. *p < 0.01 between control and Amitriptyline-treated cells. Actin was used as loading control. a*p* < 0.01 between Amitriptyline and Amitriptyline (Amit)+Leupeptin (Leup) treated cells.

Previous studies have shown that cathepsins and others lysosomal enzymes are relocalized to the cytosol by lysosomal destabilization during apoptosis induced by oxidative stress [17, 18]. Therefore, we hypothesized that lysosomal proteases such as cathepsin B may play an important role in amitriptyline-induced cell death through its release from the lysosomal/autophagolysosomal compartment to the cytosol. In healthy untreated HepG2 cells, cathepsin B signal colocalized with the lysosomal/autophagolysosomal marker LAMP-1 indicating that was located within lysosomes/autophagolysosomes (Figure 2A and 2B). However, in amitriptyline-treated HepG2 cells for 12 hours, the cathepsin B signal was diffuse through the cytosol and did not colocalized completely with the LAMP-1 marker suggesting lysosome/autophagolysosome membrane permeabilization (Figure 2A and 2B). Colocalization was quantified by scoring the number of positive puncta for both cathepsin B and LAMP-1.

Lysosomal/autophagolysosomal membrane permeabilization (LMP) was also assessed by acridine orange staining and quantifying the red fluorescence (when it is present in acidic vacuoles such as lysosomes and autophagolysosomes) and green fluorescence (when it is present in the cytosol and the nucleus). As is shown in Figure 2C and 2D, amitriptyline-treated cells manifested reduced red fluorescence and increased green fluorescence and a notably reduction in the red/green signal ratio suggesting lysosome/autophagolysosome membrane permeabilization [19]. To verify our results, we evaluated if the pharmacologic inhibition of cysteine, serine and threonine peptidases by leupeptin was able to inhibit amitriptyline-induced apoptosis in HepG2 cells. As is shown in Figure 2E and 2F, leupeptin treatment significantly abolished amitryptyline-induced apoptosis. Likewise, leupeptin treatment prevented caspase activation and BECLIN 1 cleavage (Figure 2F). These findings suggest that autophagolysosome permeabilization and release of lysosomal proteases such as cathepsin B may play an essential role in the switch of autophagy to apoptosis in amitriptyline treatment. Lysosome/ autophagolysosome permeabilization after Amitriptyline exposure seems to be a non-selective process because the treatment with a more selective protease inhibitor E-64d (100 μM) which specifically inhibits cysteine proteases did not prevent cell death (Figure 2F).

Lysosome/Autophagolysosome permeabilization was also confirmed by the detection of galectin puncta at leaky autophagolysosomes as it has been described that galectin translocation to phagosomal and lysosomal membranes is a marker of vacuole lysis and/or permeabilization [20, 21] (Supplementary Figure 4).

### Amitriptyline induces early autophagy

To further examine the initial autophagy activation in Amitriptyline treated HepG2 cells, we analyzed the expression of lysosomal enzymes such as β-galactosidase and cathepsin B, and proteins involved in autophagic processes such as the autophagosome markers Atg12- ATG5 and LC3.

**Figure 3:**
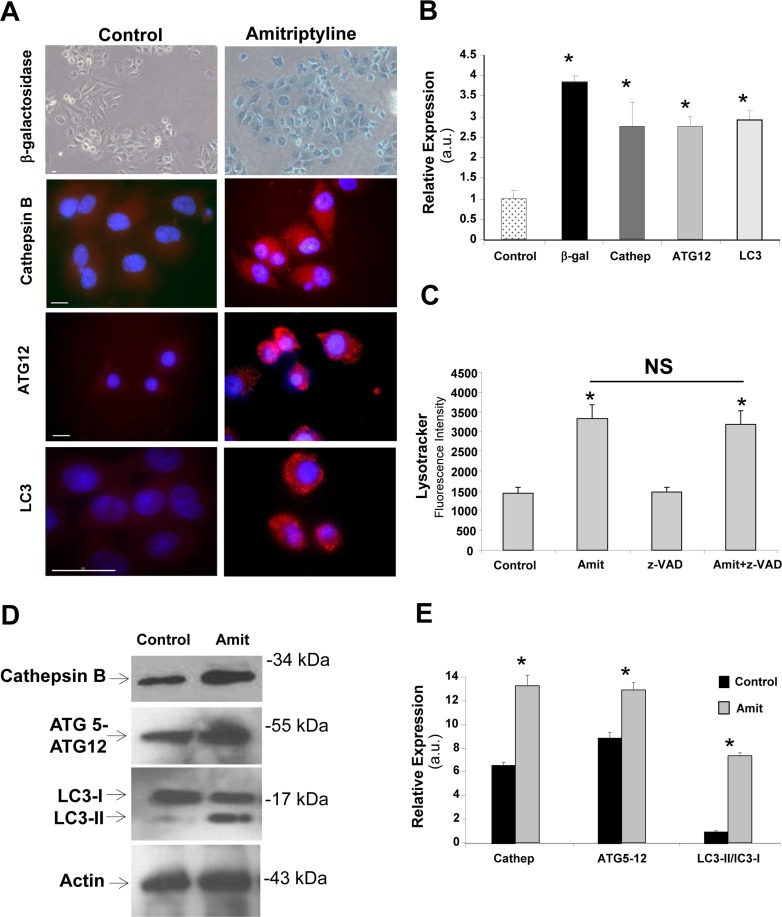
Autophagic markers in Amitriptyline-treated cells **A.** Representative images of autophagic markers Lysotracker, ATG12-ATG5, LC3, cathepsin B (Cathep) in control and 50 μM Amitriptyline-treated cells (Amit) for 6 hours that were visualized by fluorescence microscopy as described in Material and Methods. β-galactosidase (β-gal) staining was examined by light microscopy as described in Material and Methods. Bar = 15 μm. **B.** Quantification of autophagic markers expression levels was performed by image analysis using the ImageJ software. **C.** Quantification of acidic vacuoles in control and in cells treated for 6 hours with Amitriptyline and Amitriptyline+z-VAD by Lysotracker staining and flow cytometry analysis. **D.** Protein expression levels of Cathepsin B, Atg12-ATG5 and LC3 in control and 50 μM Amitriptyline-treated cells for 6 hours were performed by Western blotting as described in Material and Methods. Actin was used as loading control. **E.** Densitometry of Cathepsin B, Atg12-ATG5 and LC3 Western blotting. Data represent mean±SD of 3 separate experiments. *p < 0.01 between control and Amitriptyline-treated cells. NS = not significant.

Figure 3A and 3B show that lysosomal enzymes and autophagic proteins were overexpressed after 6 hours of Amitriptyline treatment (before apoptosis switch at 12 hours) both by immunofluorescence and Western blot analysis. The amount of β-galactosidase in Amitriptyline- treated HEPG2 cells was approximately 4 fold higher that in controls (Figure 3A and 3B). Likewise, there was a significant 2.5 fold increase in cathepsin B expression in Amitriptyline-treated HepG2 cells (Figure 3A and 3B). Furthermore, autophagic markers such as Atg12-ATG5 and LC3 were also up-regulated (Figure 3A and 3B). The number of LC3 dots per cell was 7±3.85 in control cells *versus* 65±12.86 in cells treated with Amitriptyline (*n* = 20, *p* < 0.001). In addition, we found much greater levels of acidic vacuoles after 6 hours of Amitriptyline treatment by using LysoTracker staining coupled with flow cytometry analysis, suggesting increased lysosomes/ autophagolysosomes in HepG2 treated cells (Figure 3C). Amitriptyline-induced increase in acidic vacuoles was not significantly reduced in the presence of the caspase inhibitor z-VAD suggesting that caspases were not involved in autophagic activation.

Increased cathepsin B levels in Amitriptyline- treated HepG2 cells was also confirmed by Western blotting (Figure 3D and 3E). Additionally, protein levels of ATG12-ATG5 and BECLIN 1 were also increased in Amitriptyline-treated HepG2 cells (Figure 3D and 3E). To verify autophagy activation in Amitriptyline-treated HepG2 cells, we also investigated the conversion of LC3-I to LC3-II as a marker of autophagic activity and autophagosomes number by Western blotting. We found a significant increase in LC3-II conversion in Amitriptyline- treated cell extracts indicating enhanced autophagosome formation or accumulation (Figure 3D and 3E).

### Amitriptyline induces mitophagy

**Figure 4:**
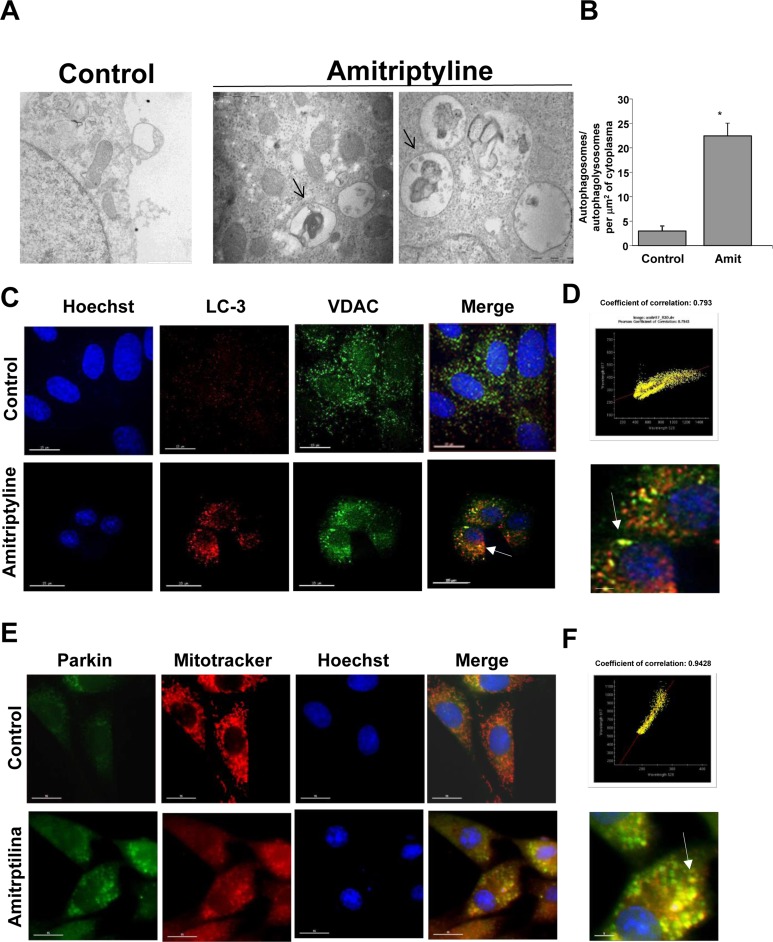
Mitophagy in Amitriptyline-treated cells **A.** Ultrastructure of Amitriptyline-treated cells. Control HepG2 cells showed mitochondria with typical ultrastructure. Bar = 1 μm. Laminar bodies and autophagosome with mitochondria-like organelles were present in Amitriptyline-treated cells (black arrows); Bar = 0.5 μm. **B.** Quantification of the number of autophagosomes and autophagolysosomes per 10 μm2 of cytoplasm were scored in 50 cells. Data are presented as means±SD. **C.** HepG2 cells were incubated in the presence or absence of 50 μM Amitriptyline for 6 h. Then, cells were fixed, and immunostained with anti-LC3 (autophagosome marker) and VDAC (mitochondrial marker) and examined in a fluorescence microscope as described in Material and Methods. **D.** Colocalization of both markers (white arrows) was assessed by the DeltaVision software. Merged high-magnification image is shown. Bar = 15 μm. **E.** Parkin translocation to mitochondria in HepG2 cells treated with Amitriptyline for 6 hours. Cells were stained with Mitotracker (red) and anti- Parkin (green). Parkin translocation to small round-shape depolarized mitochondria (red arrow). **F.** Colocalization analysis (coefficient of correlation) of Parkin and depolarized mitochondria was assessed by the DeltaVision software. Merged high-magnification image is shown. Bar = 15 μm. *p < 0.01 *vs*. control.

To date, one of the most convincing and standard method to detect autophagy is to examine the ultrastructure of cells by transmission electron microscopy [22]. Therefore, we examined the ultrastructural morphology of HepG2 cells after Amitriptyline treatment. Figure 4A shows the typical autophagic features of HepG2 cells after treatment with 50 μM Amitriptyline for 6 hours whereas untreated cells had normal nuclear and cytoplasmic morphology. In summary, vacuoles (arrows) appeared in the cytoplasm, and these membrane compartments contained mitochondria-like organelles. The quantification of autophagosomes/autophagolysosomes per cell is shown in Figure 4B. To verify the hypothesis that Amitriptyline induces selective mitophagy in HepG2 cells, we performed a double staining immunofluorescence of LC3, an autophagosome marker and VDAC, a mitochondrial marker. Figure 4C and 4D clearly shows that LC3 colocalized with VDAC, suggesting that mitochondria were being degraded within autophagosomes in Amitriptyline-treated HepG2 cells. Colocalization was addressed before apoptosis switch and in cells in which no sign of apoptosis was observed. Extensive mitophagy by Amitriptyline treatment was also demonstrated in two additional tumor cell lines, H460 and MCF7 (Supplementary Figure 5).

### Parkin dependent mitophagy in Amitriptyline treated cells

Overwhelming evidences in mammalian cell lines have implicated mitochondrial depolarization and Parkin traslocation in mitophagy. [23]. Therefore, we examined mitochondrial depolarization and Parkin mitochondrial recruitment in Amitriptyline HepG2 treated cells. As shown in Figure 4E and 4F, Amitriptyline treatment induced mitochondrial depolarization and Parkin translocation in HepG2 cells. To explore the essential role of Parkin in mitophagy activation in Amitriptyline treated cells, we used the lentiviral delivery of Parkin- directed shRNA for the knockdown of Parkin. As shown in Supplementary Figure 6, Parkin was very efficiently silenced using specific lentivirus particles, and this sensitized toward apoptosis. These data suggest that perturbing mitophagy by silencing Parkin led to enhanced apoptotic signaling.

**Figure 5:**
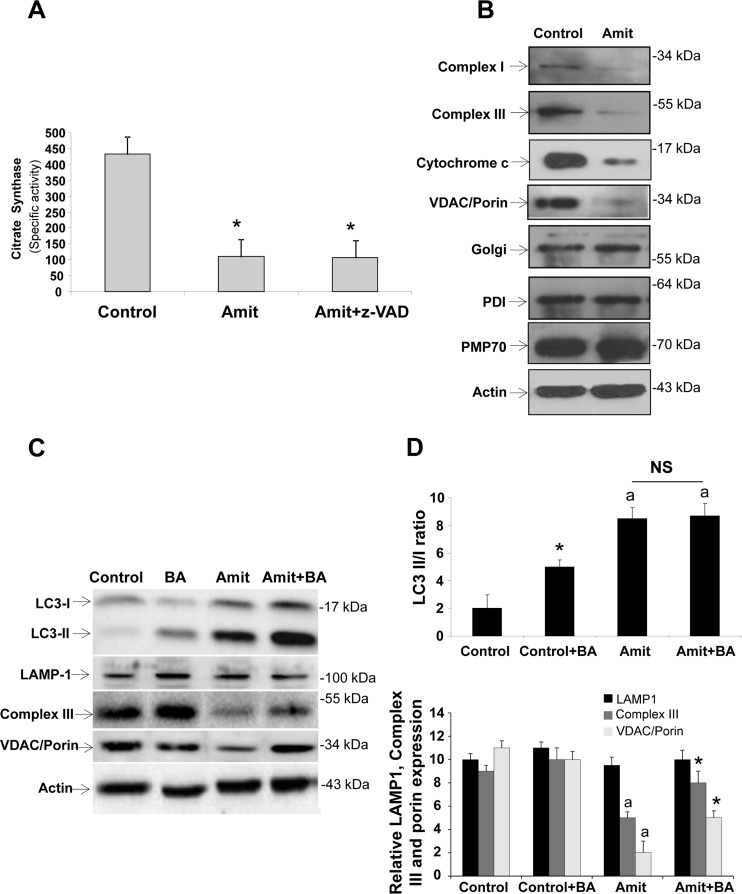
Mitophagy and autophagic flux in Amitriptyline-treated cells **A.** Mitochondrial mass measured by citrate synthase specific activity in control and Amitriptyline-treated (Amit) HEPG2 cells. **B.** Western blot analysis of mitochondrial (complex I, 30 kDa subunit; complex III, core 1 subunit; and VDAC/Porin), Golgi (Golgi marker), endoplasmic reticulum (PDI), and peroxisome (catalase) proteins in HepG2 cells treated with Amitriptyline (50μM) for 12 h. Actin was used as loading control. **C.** Autophagic flux. Determination of LC3B II, LAMP-1, complex III (core 1 subunit) and VDAC/Porin expression levels in the presence and absence of 20 nM bafilomycin A1 (BA) for 6 h in HepG2 cells treated with Amitriptyline (50μM) for 12 h. Total cellular extracts were analyzed by Western blotting. Actin was used as a loading control. **D.** Densitometry of LC3B II, LAMP-1, complex III (core 1 subunit) and VDAC/Porin. Data represent mean±SD of 3 separate experiments. *p < 0.01 *vs*. no bafilomycin A1 treatment; a*p* < 0.01 *vs*. control. NS = not significant.

Selective mitophagy and mitochondrial mass depletion in Amitriptyline treated HepG2 cells were also confirmed by measuring citrate synthase activity, as a marker of mitochondrial mass, and the expression levels of mitochondrial proteins. Amitriptyline induced a remarkably reduction in citrate synthase activity (Figure 5A) and protein expression levels of complex I (30 kDa subunit), complex III (core 1 subunit), VDAC/Porin and cytochrome c (Figure 5B and Supplementary Figure 7). In contrast, the expression of Golgi (Golgi marker), endoplasmic reticulum (PDI, protein disulfide isomerase) and peroxisome (PMP70) markers were not affected compared to controls (Figure 5B and Supplementary Figure 7). These results suggest that Amitriptyline specifically induces the selective degradation of mitochondria.

### Autophagic flux is impaired in Amitriptyline- exposed HepG2 cells

To assess autophagic flux, we determined LC3B- II levels after Amitriptyline treatment in combination with the proton ATPase inhibitor bafilomycin A1 (BA) (20 nM). As expected, BA treatment in control HepG2 cells led to a significant increase in the amount of LC3-II, suggesting that autophagic flux was normal in control HepG2 cells (Figure 5C and 5D). However, BA treatment in Amitriptyline-treated HepG2 cells increased slightly but not significantly LC3-II expression levels (Figure 5C and 5D), indicating that autophagic flux was impaired in Amitriptyline-treated HepG2 cells.

To confirm increased mitochondrial degradation, Complex III (core 1 subunit) and VDAC/Porin expression levels by Western blotting were also checked after BA treatment. Results showed that inhibition of autophagosome-lysosome fusion increased Complex III (core 1 subunit) and VDAC/Porin levels, but not LAMP-1, suggesting specific delivery and degradation of mitochondria within autophagolysosomes in Amitriptyline-treated HEPG2 cells.

### Mitophagy by Amitriptyline treatment is initially pathological or protective?

To study whether mitophagy initially promotes cell protection or plays a role in cell demise, we pharmacologically inhibited autophagosomal formation using 3-methyladenine (3MA), a class III PtdIns3K inhibitor. Inhibition of autophagy increased apoptosis suggesting that mitophagy may initially acts as a mechanism of cell protection in this model (Supplementary Figure 8A).

To further verify the initial protective role of autophagy in apoptosis induction under Amitriptyline treatment, apoptosis was examined in Amitriptyline treated wild-type and Atg5−/− MEFs. Amitriptyline induced low levels of apoptosis in wild-type MEFs as determined by detecting caspase activation, cytochrome c release, and nuclear condensation (Supplementary Figure 8B). In contrast, Atg5−/− MEFs were significantly more sensitive to amitriptyline treatment with a significant 1.6-fold increase in apoptosis over that in wild-type cells (Supplementary Figure 8B).

### Amitriptyline specifically inhibits mitochondrial complex III activity

**Figure 6:**
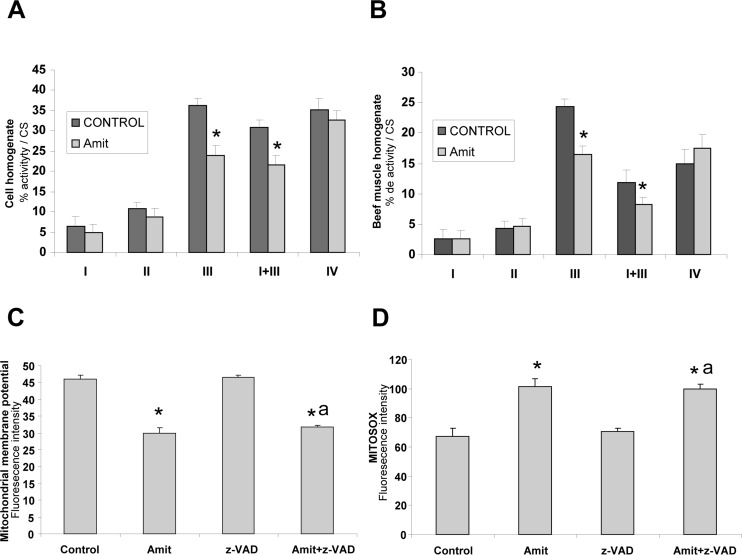
Mechanism of action of Amitriptyline: mitochondrial dysfunction **A.** Mitochondrial enzyme activities of respiratory chain complexes in HepG2 cell treated with Amitriptyline (Amit) (50 μM) for 6h. NADH:coenzyme Q1 oxidoreductase (complex I), ubiquinol:cytochrome *c* oxidoreductase (complex III), NADH: cytochrome c reductase (complex I+III); cytochrome *c* oxidase (complex IV), and citrate synthase (CS) were determined as described in Material and Methods. Results (mean±SD) are expressed in U/CS (units per citrate synthase). **B.** Mitochondrial enzyme activities of respiratory chain complexes in bovine muscle homogenates in the presence of Amitriptyline (50 μM) for 6 hours. **C.** Mitochondrial depolarization in Amitriptyline-treated HepG2 cells for 6 hours. Membrane potential was assessed by flow cytometry using MitoTracker Red. A clear decrease of mitochondrial potential was observed Amitriptyline-treated cells. **D.** ROS generation in Amitriptyline-treated HepG2 cells for 6 hours. Cellular generation of ROS in HepG2 cells was determined by flow cytometry using MitoSox, as described in Materials and Methods. *p < 0.01 between control and Amitriptyline-treated cells.

To determine the presence of mitochondrial dysfunction in Amitriptyline-treated cells, we measured the activities of the respiratory chain enzymes in control and treated cells (Figure 6A). Treated cells showed a significant reduction in the activity all of the mitochondrial respiratory chain complexes when referred to cell protein concentration in agreement with the presence of extensive mitophagy (data not shown). However, only activities of complexes III and I+III, were significantly reduced when activities of mitochondrial complexes were referred to citrate synthase, a marker of mitochondrial mass (Figure 6A). These results suggest that Amitriptyline specifically acts at the level of complex III.

To verify that Amitriptyline specifically inhibits complex III activity, we examined the effect of Amitriptyline on standard mitochondrial complex activities in bovine muscle homogenates. Amitriptyline caused a statistically significant decrease of complex III activity in bovine muscle homogenates, but had no effect on others mitochondrial complexes (Figure 6B).

To assess the functional consequences of reduced complex III activity in Amitriptyline-treated cells, we determined ΔΨ_m_ in both control and amitriptyline treated-HepG2 cells by flow cytometry. As shown in Figure 6C, Amitriptyline-treated cells showed a significant reduction in ΔΨ_m_ that was independent of caspase activation. It is well established that mitochondrial dysfunction is associated with an induction of ROS production in mitochondria. Therefore, we examined ROS levels in control and Amitriptyline-treated cells by flow cytometry using MitoSox^™^, a red fluorescent mitochondrial superoxide indicator. There was an approximately two fold increase in ROS production in Amitriptyline-treated cells that was also independent of caspase activation (Figure 6D). MitoSox signal strongly co-localized with LC3-marker consistent with increased ROS production in mitochondria that are extensively engulfed by autophagosomes (Supplementary Figure 9).

### Antioxidants protect against oxidative stress induced by Amitriptyline treatment

**Figure 7:**
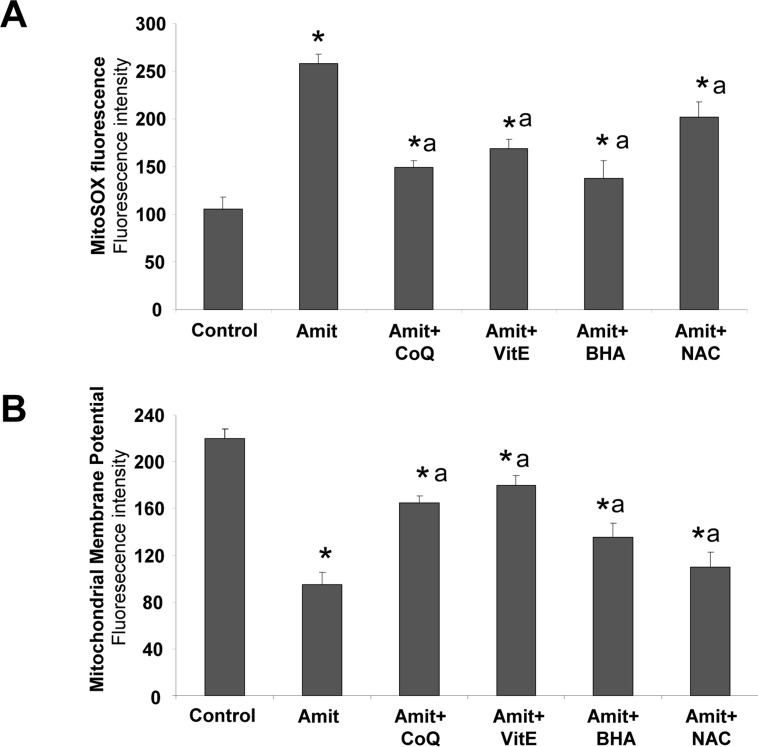
Antioxidants alleviate Amitriptyline-induced mitochondria dysfunction HepG2 cells were cultured for 6 h with 50μM Amitriptyline (Amit) in the presence of CoQ, Vitamin E, BHA or NAC. **A.** ROS production was analyzed at 6 h by Mitosox staining and flow cytometry as described in the Materials and methods section. **B.** Mitochondrial membrane potential was analyzed at 6 h by flow cytometry using MitoTracker Red. Data represent the mean±SD of three separate experiments. *P < 0.01, between control and Amitriptyline treated cells; ^a^*p* < 0.01, between the absence or presence of antioxidant.

To further examine the role of ROS generation on Amitriptyline-induced autophagy, we cultured control and Amitriptyline-treated HepG2 cells in the presence of four antioxidants and examined ROS production, mitochondrial depolarization, the rate of autophagy and cell death. The presence of ROS scavengers coenzyme Q_10_ (CoQ), Vitamin E (VitE), butylated hydroxyanisole (BHA) and N-acetylcysteine (NAC) significantly reduced ROS generation following Amitriptyline treatment in HepG2 cells (Figure 7A).

Interestingly, antioxidant treatment restored ΔΨm (Figure 7B) as well as Parkin translocation, autophagolysosome/lysosome permeabilization and cell death (Supplementary Figure 10). Together these observations suggest that ROS production caused by mitochondrial dysfunction may be involved in triggering in Amitriptyline-induced early and extensive mitophagy and subsequent apoptosis in HepG2 cells.

### Amitriptyline cytotoxicity in HepG2 cells and primary human hepatocytes

**Figure 8:**
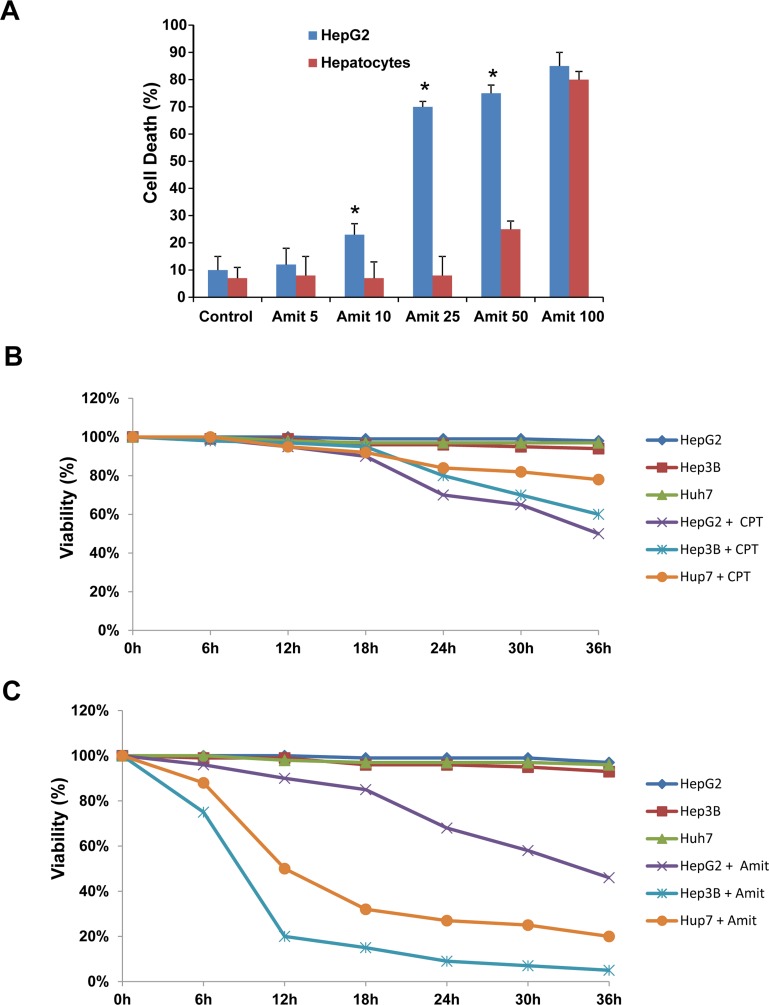
Effect of Amitriptyline on human hepatocytes and p53 −/− cell lines **A.** Amitriptyline (Amit) toxicity on normal human hepatocytes and HepG2 cells. Cells were seeded in six-multiwell plates, at a density of 100,000 cells/well. After 24 h of culture, serial concentrations of Amitriptyline (0, 5, 10, 25, 50 and 100 μM) were added to the culture medium and cells were further incubated for 24h. Cells were then harvested and viability was analyzed by using the vital dye exclusion assay as described in the Materials and methods section. **B.** Camptothecin CPT (10 μM) toxicity on HepG2, Hep3B and Hup7 cell lines. **C.** Amitriptyline (50 μM) toxicity on HepG2, Hep3B and Hup7. *p < 0.01, between HepG2 and hepatocytes.

To explore the specificity of Amitriptyline cytotoxicity on cancer cells, we examined its effects on the rate of apoptosis with flow cytometry in HepG2 cells and human hepatocytes. HepG2 and human hepatocytes were treated with 0, 5, 10, 25, 50 and 100 μM of Amitriptyline for 24 h and cell death was determined (Figure 8A). Amitriptyline showed a greater ability to induce cell death of cultured HepG2 when compared to primary cultured human hepatocytes at concentrations of 10, 25 and 50 μM.

### Amitriptyline-induced cell death was independent of p53 status

As drug-induced cell death is dependent on the tumor suppressor protein p53 in hepatoma cells [24], we next assessed the effectiveness of Amitriptyline respect to a standard genotoxic treatment with the topoisomerase I inhibitor camptothecin (CPT) in three hepatoma cell lines with different p53 status: HepG2 with wild-type p53, Huh 7 with mutated p53 and Hep3B with non-sense mutation on p53 gene. Irrespective of mutant or wild-type p53 status, CPT induced similar levels of DNA damage in the hepatome cell lines as assessed by detecting phosphorylated histone H2A.X (gamma-H2AX) foci, a biomarker of double stranded breaks in DNA (data not shown).

As is shown in Figure 8A and 8B, hepatoma cells lines with mutated or absent p53 exhibited marked resistance to cell death by CPT treatment, whereas were notably sensitive to Amitriptyline treatment. Therefore, Amitriptyline-induced cell death was p53-independent and can be an effective treatment for p53 mutant tumors.

## DISCUSSION

In the present study, we show that Amitriptyline treatment activates early and extensive mitophagy in HepG2 cells that eventually leads to apoptosis. Amitriptyline treatment in HepG2 cells induces mitochondrial dysfunction characterized by reduced ∆Ψm, increased ROS production and mitophagy activation. Our results support the hypothesis that Amitriptyline and related compounds exert their effect *via* inhibition of complex III of the mitochondrial respiratory chain [25].

The rationale for targeting mitochondria for therapeutic applications is based on the knowledge that this organelle plays a critical role in the regulation of energy metabolism, ROS production and apoptosis [26]. There are evidences that suggest that cancer cells compared to normal cells exhibit increased intrinsic ROS stress associated with oncogenic transformation, increased metabolic activity and mitochondrial dysfunction [27, 28]. Malfunction of mitochondria also alters cellular apoptotic response to anticancer agents [28]. On the other hand, the majority of tumor cells frequently possess very little antioxidative enzymes such as catalase, superoxide dismutase and glutathione peroxidase that makes tumor cells very vulnerable to oxidative stress. Consequently, strategies for increasing ROS levels has been proposed as a way to selectively kill cancer cells without causing significant toxicity to normal cells [29, 30]. Therefore, Amitriptyline may fit properly into the new anticancer strategy named *oxidative therapy*.

In this study, we show evidences that Amitriptyline induces selective autophagy of damaged mitochondria preceding apoptosis activation. The mitochondrion is one of the organelles that can become target for autophagic degradation in a process known as mitophagy [31, 32]. Recent works have explored how the maintenance of a functional mitochondrial network is mediated by Parkin, which is an E3 ligase that selectively recognize and eliminate damaged mitochondria [33]. Amitriptyline- induced mitophagy was assessed using several approaches. We studied LC3-II expression levels, the only protein known to be specifically localized to autophagic structures throughout the entire autophagic process [34]. Nevertheless, it is important to point out that increased LC3-II levels have been associated not only with an enhanced autophagosome synthesis but also with a reduced autophagosome turnover. Thus, autophagic flux appeared to be also impaired after Amitriptyline treatment suggesting that increased LC3-II levels after Amitriptyline treatment may be caused by both an initial enhanced autophagosome synthesis and subsequent autophagic flux impairment.

Autophagy is related to cell death by apoptosis, but this relationship is still not well understood. In adddition, pharmacological or genetic inhibition of autophagy enhances the proapoptotic action of Amitriptyline. A complex relationship between autophagy and apoptosis has been suggested for several xenobiotics that induced both processes (for example, imiquimod in basal cell carcinoma [35] or efavirenz in hepatic cells [36]; in both cases the inhibition of autophagy promoted apoptosis which is congruent with our results). One of the mechanisms of interaction between autophagy and apoptosis is the possibility that lysosomal enzymes can activate apoptosis [37]. It has been hypothesized that lysosomal enzymes release through membrane permeabilization of lysosomes/autophagolysosomes may promote apoptosis by cleaving a variety of substrates, including members of the Bcl-2 protein family, PARP1, sphingosine kinase 1, XIAP and caspases [38]. Supporting this hypothesis, our results show that after Amitriptyline treatment, cathepsin B is relocalized from the lysosomes/autophagolysomes to the cytosol and that acridine orange up-take and retention by acidic vacuoles is impaired. Furthermore leupeptin a cysteine, serine and threonine proteases inhibitor reduced significantly Amitryptyline- induced cell death by apoptosis.

Autophagolysosome membranes can be particularly prone to ROS-mediated damage, because mitochondria degradation can increase autophagolysosome iron content which can produce increased ROS production through the Fenton reaction [39]. Thus, enzymatic or oxidative modifications of membrane lipids affect the membrane permeability, which can lead to autophagolysome permeabilization. Therefore, in accordance with our results, antioxidants may prevent both mitochondrial and autophagolysosome ROS production and subsequent permeabilization.

Once activated, caspases can cleave autophagy- associated protein BECLIN 1 abrogating its pro- autophagic effect and sensitize cells to apoptosis [40, 41]. In agreement with this hypothesis, our results show that Amitriptyline induce caspase-dependent cleavage of BECLIN 1.

Our finding also show that Amitriptyline treatment can be an effective therapeutic approach for cancer malignancies with mutated or absent p53 which are resistant to conventional genotoxic treatments.

In conclusion, Amitriptyline induces early mitochondrial dysfunction and extensive mitophagy in HepG2 cells (Supplementary Figure 11). Activation of this process promotes cell survival but exceeding a certain threshold of mitochondrial dysfunction and mitophagy activation is associated with autophagic stress, autophagolysosome permeabilization and cell death by apoptosis.

Targeting mitochondria by Amitriptyline treatment can be an alternative therapeutic approach for cancer malignancies, particularly for those with mutated or absent p53 which are commonly resistant to conventional genotoxic treatments. Further research in xenograft models is warranted to confirm these findings and to assess the safety profile of Amitriptyline for this indication.

## MATERIALS AND METHODS

### Reagents

Monoclonal Anti-Actin antibody, rabbit anti- VDAC1/Porin, rabbit anti-BECLIN 1 and E-64d were obtained from Sigma-Aldrich (St. Louis, MO, USA). Monoclonal antibodies against complex III (core 1 subunit) and complex I (30 kDa subunit), MitoSox Red, CMH2-DCFDA, 10-N-nonyl acridine orange (NAO), Acridine orange, MitoTracker, LysoTracker red DND- 99 and Hoechst 3342 were obtained from Invitrogen/ Molecular Probes (Eugene, OR, USA). Anti-cytochrome c antibody was obtained from BD Biosciences Pharmingen (San Jose, CA, USA) and anti-GAPDH monoclonal antibody (clone 6 C5) was from Calbiochem-Merck Chemicals Ltd. (Nottingham, UK). Anti-hATG12-ATG5 was obtained from Biosensis (South Australia, Australia). Anti-MAP LC3 (N-20), anti-catalase (H-300), anti-PDI (H-160), anti-Cathepsin B, anti-Bcl-x, anti-Bcl-2, anti- Bax, anti-Mcl-1, anti-survivin, anti-galectin-3 and anti- LAMP-1 were obtained from Santa Cruz Biotechnology (Santa Cruz, CA, USA). Anti-Golgi marker (58K-9) was purchased from Abcam (Cambridge, UK). Anti-caspase 3 and Anti-active caspase 3 was obtained from Cell Signaling Technology (Beverly, MA, USA). Pancaspases inhibitors z-VAD-fmk and z-DEVD-fmk were from R&D Systems (Minneapolis, MN, USA). Protease inhibitors were obtained from Boehringer Mannheim (Indianapolis, IN, USA). The Immun Star HRP substrate kit was from Bio-Rad Laboratories Inc. (Hercules, CA, USA). All other chemicals were purchased from Sigma-Aldrich.

### Cell culture

Hepatoma cell lines with different p53 genetic profile (HepG2, Huh7 and Hep3B) were cultured in D-MEM medium (4500 mg/L glucose, L-glutamine, piruvate; Invitrogen) supplemented with 20% fetal bovine serum (FBS, Linus) and antibiotics (Sigma Chemical Co., St. Louis, MO, USA). Cells were incubated at 37°C in a 5% CO_2_ atmosphere. Amitriptyline (Sigma Chemical Co., St. Louis, MO, USA) used for treatments of cell cultures was diluted with fetal bovine serum. The Hep3B cell line is p53 null; the HepG2 a wild-type p53 gene, while the HuH7 cell line carry p53 point mutations.

### Human hepatocytes

Primary human hepatocytes were isolated by the two-steps collagenase perfusion following the procedure decribed by Pichard et al. [42] The liver biopsies were obtained from patients submitted to hepatic resections from colorectal metastasis after obtained written consent. Samples were negative for human immunodeficiency and hepatitis viruses. The protocol has been approved by the Ethical Committee of the Hospital Universitario “Virgen del Rocío” of Sevilla (Spain).

### Atg5−/− mouse fibroblasts

Fibroblasts (MEFs) derived from wild-type and Atg5−/− mouse embryos were a kind gift of Noboru Mizushima, Tokyo Medical and Dental University, Japan [43].

### Treatments

Cells were cultured with 50 μM Amitriptyline in the absence or presence of 30 μM CoQ_10_ or 30 μM vitamin E (VitE) or Butylated hydroxyanisole (BHA) or N-acetylcysteine (NAC) for 24 h. Amitriptyline used for treatments of cell cultures was diluted with fetal bovine serum.

### Proliferation rate assay and vital counts

Two hundred thousand cells were cultured with Amitriptyline (50 μM), in the absence or presence of z-VAD-fmk (100 μM) or z-DEVD-fmk (100 μM) for 24 h. Cell counting was performed from 3 high power fields using an inverted microscope and a 40X objective. Trypan blue vital count was performed to assess cell viability.

### Analysis of apoptosis

Apoptotic cells were identified either by fluorescence microscopy or by flow cytometry. Thus, apoptosis was assessed by observing nuclei fragmentation by Hoechst staining, cytochrome c release and caspase 3 activation by fluorescence microscopy. Viable cells were determined from their normal cell and nuclear morphology and exclusion of propidium iodide. In each case, 10 random fields and > 500 cells were counted in each experiment.

Phosphatidylserine (PS) translocation from the inner to the outer leaflet of the plasma membrane is one of the earliest apoptotic features. We used the PS- binding protein Annexin V conjugated with FITC to identify PS exposure in HepG2 cells by flow cytometry. The binding of annexin V to cell surface PS was detected with a commercial Annexin Apoptosis Kit (Santa Cruz Biotechnology) according to the manufacturer's instructions. To distinguish cells that had lost membrane permeability, propidium iodide (PI) was added to a final concentration of 10 mg/ml immediately before analysis by flow cytometry.

### Mitochondrial ROS production

Mitochondrial ROS generation was assessed by MitoSox™, a red mitochondrial superoxide indicator. Approximately 1 × 106 cells were incubated with 1μM MitoSox for 30 min at 37°C, washed twice with PBS and resuspended in 500 μl of PBS and analyzed by flow cytometry.

### Mitochondrial membrane potential (ΔΨm)

Cells were cultured in six-well plates (35 mm diameter well) and, at confluence were treated with drugs 50 μM Amitriptyline in the absence or presence of membrane antioxidants. After 24 h, 100nM Mitotracker was added and the incubation was prolonged for 30 min. Once the incubation was finished, cells were harvested, incubated with fresh medium, washed, centrifuged (500xg), resuspended in DMEN medium and analyzed by flow cytometry.

### Mitochondrial respiratory chain enzyme activities

Activities of NADH:coenzyme Q1 oxidoreductase (complex I), succinate dehydrogenase (complex II), cytochrome c oxidase (complex IV), ubiquinol:cytochrome c oxidoreductase (complex III), NADH:cytochrome c reductase (complex I+III), and citrate synthase (CS) were determined in sonicated permeabilized hepatoma cells and bovine muscle homogenates using previously described spectrophotometric methods [44]. Results are expressed as units per CS. Proteins of cell homogenates were analyzed by the Lowry procedure [45].

### Electron and fluorescence microscopy

Electron and fluorescence microscopy were performed using protocols previously described by our group [46]. In fluorescence microscopy, colocalization of immunofluorescense markers was assessed by calculating Pearson's correlation coefficient by DeltaVision software.

### Western blotting analysis

Whole cellular lysates were prepared in a buffer, gentle shaking, composed of 0.9% NaCl, 20 mM Tris-HCl, pH 7.6, 0.1% triton X-100, 1 mM phenylmethylsulfonylfluoride and 0.01% anti-proteases. Electrophoresis was carried out in a 10-15% acrylamide SDS/PAGE. Proteins were transferred to Immobilon membranes (Amersham Pharmacia) and, after blocking over night at 4°C, incubated with the respective antibody solution at 1:1000 dilutions. Then, membranes were probed with their respective secondary antibody (1:2500). Immunolabeled proteins were detected by using a chemioluminiscence method (Bio-Rad). Protein concentration was determined by the Bradford method [47].

### Lysotracker red assay

LysoTracker Red DND-99 (100 nM) was added to cultured HepG2 cells for 30minutes and washed twice with fresh DMEM. Cells were then harvested and resuspended in fresh prewarmed medium, and red lysosomal fluorescence was quantified by flow cytometry.

### β-galactosidase assay

Cultured cells were washed in PBS (pH 7.4), fixed with 3.7% formaldehyde, and incubated overnight at 37°C in freshly prepared staining buffer [1 mg/ml X-gal (5-bromo-4-chloro-3-indolyl β-D-galactoside), 5 mM K_3_Fe [CN]_6_, 5 mM K_4_Fe [CN]_6_, and 2 mM MgCl_2_ in PBS (pH 6.0) or in citrate-buffered saline (pH 4.5)] [48]. At the end of the incubation, cells were washed with PBS, examined, and photographed using a Leica CTR 5000 microscope. β-Galactosidase staining was quantified using ImageJ software.

### Lysosomal/autophagolysosomal membrane permeabilization (LMP) assessment Acridine Orange (AO) staining and analysis by fluorescence microscopy

AO is a lysosomotropic metachromatic fluorochrome. When excited with blue light, AO emits red fluorescence at high concentrations (when it is present in lysosomes o cell acidic compartments) and green fluorescence at low concentrations (when it is present in the cytosol and the nucleus). Cells were seeded in six- well plates containing glass coverslips, and exposed to Amitriptyline for 24 h. Cells were then incubated with 1 mM AO for 15 min at 37°C and washed with PBS. Coverslips were mounted over glass slides with PBS and immediately observed and photographed using a fluorescence microscope (Leica DM 5000B).

### AO staining analysis by flow cytometry

Cells were exposed to Amitriptyline for 24 h. Both floating and attached cells were collected, washed with PBS and resuspended in PBS to a final concentration of 1×10^6^ cells in 1.5 ml. Cells were then incubated with 1 mM AO (or without AO to measure autofluorescence) for 15 min at 37°C.

### Cathepsin release

Cathepsin redistribution from lysosomes/autophagolysosomes to the cytosol was assessed by Immunofluorescence techniques using antibodies against cathepsins B and LAMP1 as a marker of lysosomal/autophagolysosomal compartment. In healthy cells, Cathepsin-specific immunostainings reveal cytoplasmic puncta structures that are surrounded by lysosomal/autophagolysosomal membrane proteins such as LAMP-1. After LMP, the immunofluorescence detection of Cathepsin reveals a diffuse staining throughout the entire cell.

### Galectin puncta

LMP was also detected by the galectin puncta assay as previously described [20]. Average numbers of galectin-3 puncta were quantified in at least 50 cells.

### Parkin silencing

Parkin shRNA (h) Lentiviral Particles from Santa Cruz is a pool of viral particles containing 3 target-specific constructs that encode 19-25 nt (plus hairpin) shRNA designed to reduce Parkin gene expression. Control shRNA Lentiviral Particles contains a shRNA construct encoding a scrambled sequence that will not lead to the specific degradation of any known cellular mRNA. HepG2 were infected with Parkin shRNA (h) Lentiviral Particles o control scrambled lentiviral particles according to the manufacturer's instructions. Cells were plated in a 12- well plate for 24 h before the viral infection. Cells were grown to ~50% confluence and then infected. Stable clones expressing the shRNA were selected by incubating cells with a mixture of complete medium with puromycin. Next, the medium was replaced with fresh puromycin-containing medium every 3 to 4 days until resistant colonies were identified. Colonies were expanded and assayed for Parkin silencing.

### Statistical analysis

All results are expressed as means±SD. Parametric tests (Student *t* test for comparing two groups and ANOVA for more than two groups) were used whenever assumptions (of normality and homogeneity of variances) were met.

## SUPPLEMENTARY FIGURES



## Abbreviations

Amit: amitriptyline
AO: acridine orange
BA: bafilomycin A1
BHA: butylated hydroxyanisole
CS: Citrate synthase
CoQ: Coenzyme Q10
CPT: camptothecin
≠Ψm: mitochondrial membrane potential ER, endoplasmic reticulum
ERAD: ER associated degradation system
HCC: hepatocellular carcinoma
MEFs: mouse embryonic fibroblasts
MPT: mitochondria permeability transition
3MA: 3-methyladenine
NAC: n-acetylcysteine
PDI: protein disulfide isomerase
ROS: reactive oxygen species
TEM: transmission electron microscopy
VDAC: voltage-dependent anion channel
VitE: vitamin E.

## References

[1] Jemal A, Bray F, Center MM, Ferlay J, Ward E, Forman D (2011). Global cancer statistics. CA Cancer J Clin.

[2] Perz JF, Armstrong GL, Farrington LA, Hutin YJ, Bell BP (2006). The contributions of hepatitis B virus and hepatitis C virus infections to cirrhosis and primary liver cancer worldwide. J Hepatol.

[3] Kim HR, Park HJ, Park JH, Kim SJ, Kim K, Kim J (2004). Characteristics of the killing mechanism of human natural killer cells against hepatocellular carcinoma cell lines HepG2 and Hep3B. Cancer immunology, immunotherapy: CII.

[4] Yamanaka T, Shiraki K, Sugimoto K, Ito T, Fujikawa K, Ito M, Takase K, Moriyama M, Nakano T, Suzuki A (2000). Chemotherapeutic agents augment TRAIL-induced apoptosis in human hepatocellular carcinoma cell lines. Hepatology.

[5] Wondrak GT (2009). Redox-directed cancer therapeutics: molecular mechanisms and opportunities. Antioxidants & redox signaling.

[6] Fang J, Nakamura H, Iyer AK (2007). Tumor-targeted induction of oxystress for cancer therapy. Journal of drug targeting.

[7] Ren JG, Xia HL, Just T, Dai YR (2001). Hydroxyl radical- induced apoptosis in human tumor cells is associated with telomere shortening but not telomerase inhibition and caspase activation. FEBS letters.

[8] Nguyen DM, Hussain M (2007). The role of the mitochondria in mediating cytotoxicity of anti-cancer therapies. Journal of bioenergetics and biomembranes.

[9] Bryson HM, Wilde MI (1996). Amitriptyline. A review of its pharmacological properties and therapeutic use in chronic pain states. Drugs & aging.

[10] Cordero MD, Sanchez-Alcazar JA, Bautista-Ferrufino MR, Carmona-Lopez MI, Illanes M, Rios MJ, Garrido-Maraver J, Alcudia A, Navas P, de Miguel M (2010). Acute oxidant damage promoted on cancer cells by amitriptyline in comparison with some common chemotherapeutic drugs. Anti-cancer drugs.

[11] Moreno-Fernandez AM, Cordero MD, de Miguel M, Delgado-Rufino MD, Sanchez-Alcazar JA, Navas P (2008). Cytotoxic effects of amitriptyline in human fibroblasts. Toxicology.

[12] Moreno-Fernandez AM, Cordero MD, Garrido-Maraver J, Alcocer-Gomez E, Casas-Barquero N, Carmona-Lopez MI, Sanchez-Alcazar JA, de Miguel M (2012). Oral treatment with amitriptyline induces coenzyme Q deficiency and oxidative stress in psychiatric patients. Journal of psychiatric research.

[13] Karlsson H, Gu Y, DePierre J, Nassberger L (1998). Induction of apoptosis in proliferating lymphocytes by tricyclic antidepressants. Apoptosis : an international journal on programmed cell death.

[14] Serafeim A, Holder MJ, Grafton G, Chamba A, Drayson MT, Luong QT, Bunce CM, Gregory CD, Barnes NM, Gordon J (2003). Selective serotonin reuptake inhibitors directly signal for apoptosis in biopsy-like Burkitt lymphoma cells. Blood.

[15] Lirk P, Haller I, Hausott B, Ingorokva S, Deibl M, Gerner P, Klimaschewski L (2006). The neurotoxic effects of amitriptyline are mediated by apoptosis and are effectively blocked by inhibition of caspase activity. Anesthesia and analgesia.

[16] Wu H, Che X, Zheng Q, Wu A, Pan K, Shao A, Wu Q, Zhang J, Hong Y (2014). Caspases: a molecular switch node in the crosstalk between autophagy and apoptosis. International journal of biological sciences.

[17] Roberg K (2001). Relocalization of cathepsin D and cytochrome c early in apoptosis revealed by immunoelectron microscopy. Laboratory investigation; a journal of technical methods and pathology.

[18] Roberg K, Johansson U, Ollinger K (1999). Lysosomal release of cathepsin D precedes relocation of cytochrome c and loss of mitochondrial transmembrane potential during apoptosis induced by oxidative stress. Free radical biology & medicine.

[19] Boya P, Kroemer G (2008). Lysosomal membrane permeabilization in cell death. Oncogene.

[20] Aits S, Kricker J, Liu B, Ellegaard AM, Hamalisto S, Tvingsholm S, Corcelle-Termeau E, Hogh S, Farkas T, Holm Jonassen A, Gromova I, Mortensen M, Jaattela M (2015). Sensitive detection of lysosomal membrane permeabilization by lysosomal galectin puncta assay. Autophagy.

[21] Machado FC, Cruz L, da Silva AA, Cruz MC, Mortara RA, Roque-Barreira MC, da Silva CV (2014). Recruitment of galectin-3 during cell invasion and intracellular trafficking of Trypanosoma cruzi extracellular amastigotes. Glycobiology.

[22] Gozuacik D, Kimchi A (2004). Autophagy as a cell death and tumor suppressor mechanism. Oncogene.

[23] Vives-Bauza C, Zhou C, Huang Y, Cui M, de Vries RL, Kim J, May J, Tocilescu MA, Liu W, Ko HS, Magrane J, Moore DJ, Dawson VL, Grailhe R, Dawson TM, Li C (2010). PINK1-dependent recruitment of Parkin to mitochondria in mitophagy. Proceedings of the National Academy of Sciences of the United States of America.

[24] Seitz SJ, Schleithoff ES, Koch A, Schuster A, Teufel A, Staib F, Stremmel W, Melino G, Krammer PH, Schilling T, Muller M (2010). Chemotherapy-induced apoptosis in hepatocellular carcinoma involves the p53 family and is mediated via the extrinsic and the intrinsic pathway. Int J Cancer.

[25] Daley E, Wilkie D, Loesch A, Hargreaves IP, Kendall DA, Pilkington GJ, Bates TE (2005). Chlorimipramine: a novel anticancer agent with a mitochondrial target. Biochemical and biophysical research communications.

[26] Pilkington GJ, Parker K, Murray SA (2008). Approaches to mitochondrially mediated cancer therapy. Seminars in cancer biology.

[27] Franco R, Schoneveld O, Georgakilas AG, Panayiotidis MI (2008). Oxidative stress, DNA methylation and carcinogenesis. Cancer letters.

[28] Pelicano H, Carney D, Huang P (2004). ROS stress in cancer cells and therapeutic implications. Drug resistance updates: reviews and commentaries in antimicrobial and anticancer chemotherapy.

[29] Schumacker PT (2006). Reactive oxygen species in cancer cells: live by the sword, die by the sword. Cancer cell.

[30] Trachootham D, Alexandre J, Huang P (2009). Targeting cancer cells by ROS-mediated mechanisms: a radical therapeutic approach?. Nature reviews Drug discovery.

[31] Hirota Y, Aoki Y, Kanki T (2011). Mitophagy: selective degradation of mitochondria by autophagy. Seikagaku.

[32] Kim I, Rodriguez-Enriquez S, Lemasters JJ (2007). Selective degradation of mitochondria by mitophagy. Archives of biochemistry and biophysics.

[33] Narendra D, Tanaka A, Suen DF, Youle RJ (2008). Parkin is recruited selectively to impaired mitochondria and promotes their autophagy. The Journal of cell biology.

[34] Nakatogawa H, Suzuki K, Kamada Y, Ohsumi Y (2009). Dynamics and diversity in autophagy mechanisms: lessons from yeast. Nature reviews.

[35] Huang SW, Liu KT, Chang CC, Chen YJ, Wu CY, Tsai JJ, Lu WC, Wang YT, Liu CM, Shieh JJ (2010). Imiquimod simultaneously induces autophagy and apoptosis in human basal cell carcinoma cells. The British journal of dermatology.

[36] Apostolova N, Gomez-Sucerquia LJ, Gortat A, Blas-Garcia A, Esplugues JV (2011). Compromising mitochondrial function with the antiretroviral drug efavirenz induces cell survival- promoting autophagy. Hepatology.

[37] Boya P, Gonzalez-Polo RA, Poncet D, Andreau K, Vieira HL, Roumier T, Perfettini JL, Kroemer G (2003). Mitochondrial membrane permeabilization is a critical step of lysosome- initiated apoptosis induced by hydroxychloroquine. Oncogene.

[38] Galluzzi L, Bravo-San Pedro JM, Kroemer G (2014). Organelle-specific initiation of cell death. Nature cell biology.

[39] Kurz T, Terman A, Gustafsson B, Brunk UT (2008). Lysosomes in iron metabolism, ageing and apoptosis. Histochemistry and cell biology.

[40] Wirawan E, Vande Walle L, Kersse K, Cornelis S, Claerhout S, Vanoverberghe I, Roelandt R, De Rycke R, Verspurten J, Declercq W, Agostinis P, Vanden Berghe T, Lippens S, Vandenabeele P (2010). Caspase-mediated cleavage of Beclin-1 inactivates Beclin-1-induced autophagy and enhances apoptosis by promoting the release of proapoptotic factors from mitochondria. Cell death & disease.

[41] Zhu Y, Zhao L, Liu L, Gao P, Tian W, Wang X, Jin H, Xu H, Chen Q (2010). Beclin 1 cleavage by caspase-3 inactivates autophagy and promotes apoptosis. Protein & cell.

[42] Pichard L, Raulet E, Fabre G, Ferrini JB, Ourlin JC, Maurel P (2006). Human hepatocyte culture. Methods Mol Biol.

[43] Mizushima N, Yamamoto A, Hatano M, Kobayashi Y, Kabeya Y, Suzuki K, Tokuhisa T, Ohsumi Y, Yoshimori T (2001). Dissection of autophagosome formation using Apg5- deficient mouse embryonic stem cells. The Journal of cell biology.

[44] Spinazzi M, Casarin A, Pertegato V, Salviati L, Angelini C (2012). Assessment of mitochondrial respiratory chain enzymatic activities on tissues and cultured cells. Nature protocols.

[45] Lowry OH, Rosebrough NJ, Farr AL, Randall RJ (1951). Protein measurement with the Folin phenol reagent. The Journal of biological chemistry.

[46] Rodriguez-Hernandez A, Cordero MD, Salviati L, Artuch R, Pineda M, Briones P, Gomez Izquierdo L, Cotan D, Navas P, Sanchez-Alcazar JA (2009). Coenzyme Q deficiency triggers mitochondria degradation by mitophagy. Autophagy.

[47] Bradford MM (1976). A rapid and sensitive method for the quantitation of microgram quantities of protein utilizing the principle of protein-dye binding. Anal Biochem.

[48] Lee BY, Han JA, Im JS, Morrone A, Johung K, Goodwin EC, Kleijer WJ, DiMaio D, Hwang ES (2006). Senescence-associated beta-galactosidase is lysosomal beta-galactosidase. Aging Cell.

