# Supplementary material for: Amitriptyline induces mitophagy that precedes apoptosis in human HepG2 cells
Source: Genes Cancer. 2016 Jul;7(7-8):260–77. doi: 10.18632/genesandcancer.114 (PMC5059116; doi:10.18632/genesandcancer.114)
Supplement: Supplementary file 1 [file ganc-07-260-s001.pdf]

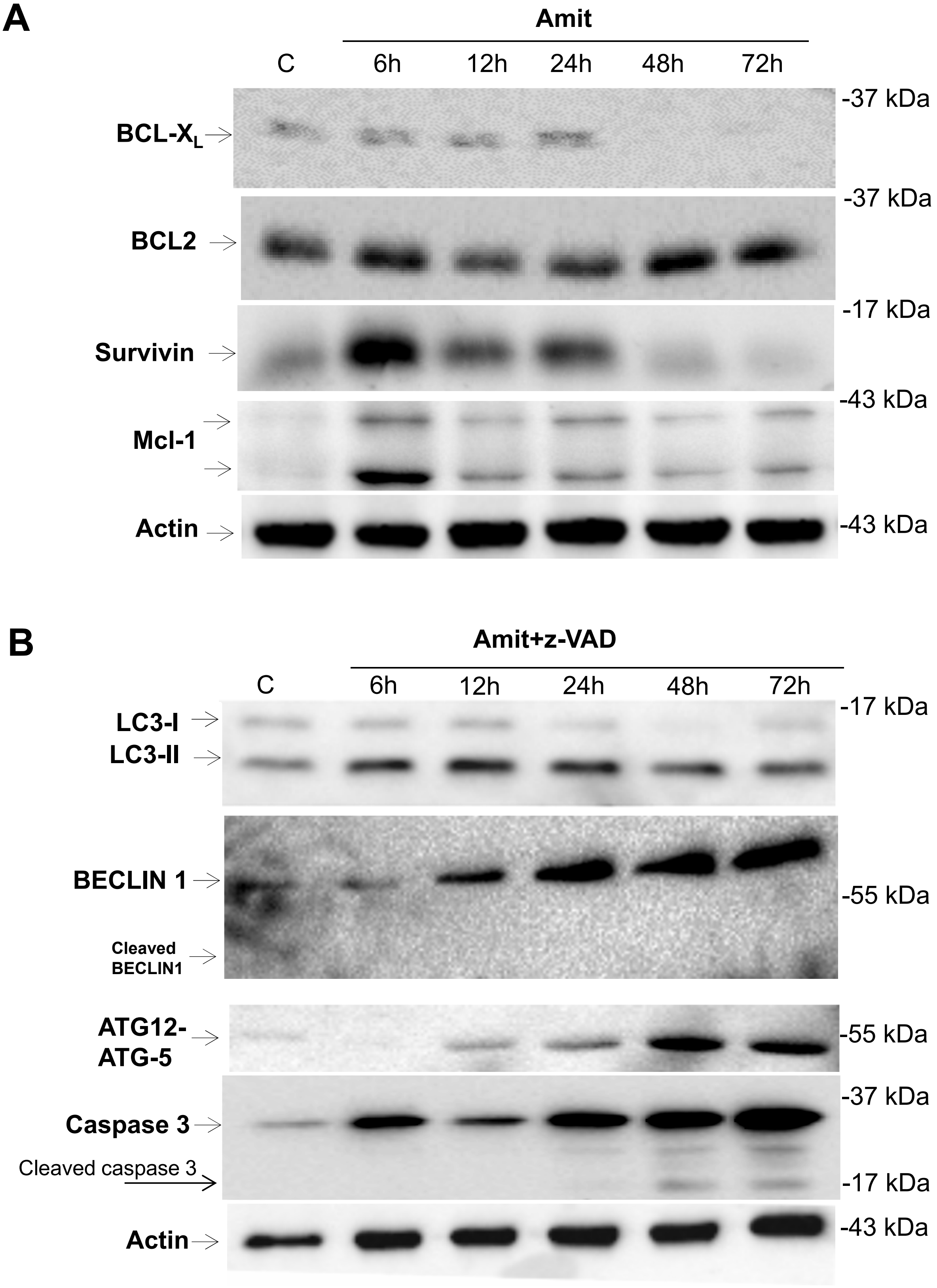

Supplementary Figure 1. (A) Anti-apoptotic protein expression levels examined by Western blotting in HepG2 cells treated with 50 μM Amitriptyline for 72h. **(B)** Amitriptyline induced caspase activation and BECLIN 1 cleavage is prevented by z-VAD. HepG2 cells were treated with 50 μM Amitriptyline in the presence of z-VAD (50 μM) for 72h. Autophagic (LC3, BECLIN 1 and ATG12-ATG5) and apoptotic (active caspase 3 and PARP) protein expression levels were examined by Western blotting. Actin was used as loading control.

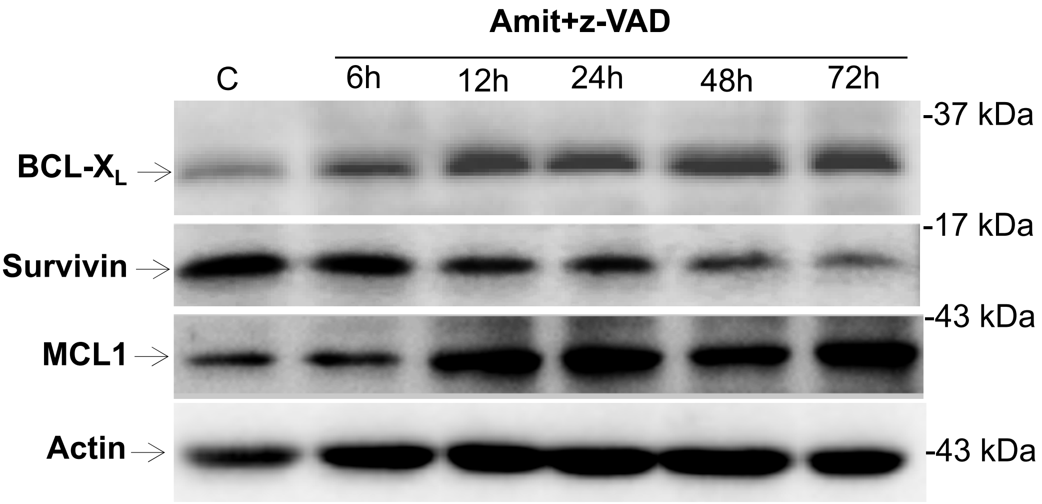

Supplementary Figure 2. Anti-apoptotic protein expression levels examined by Western blotting in HepG2 cells treated with 50 μM Amitriptyline in the presence of a caspase inhibitor. HepG2 cells were treated with 50 μM Amitriptyline in the presence of z-VAD (50 μM) for 72h. Autophagic (LC3, BECLIN 1 and ATG12-ATG5) and apoptotic (active caspase 3 and PARP) protein expression levels were examined by Western blotting. Actin was used as loading control.

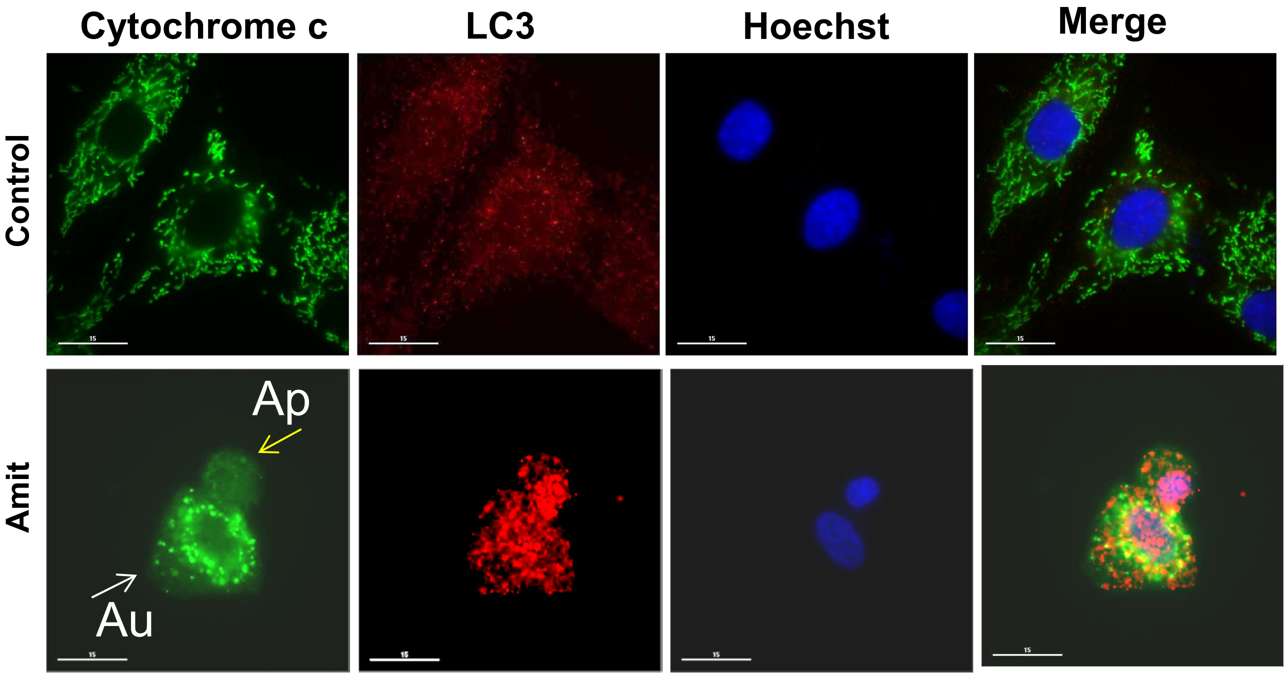

Supplementary Figure 3. Immunofluorescence microscopy. HepG2 cells were treated with 50 μM Amitriptyline for 12 hours. Then, cells were fixed and immunostained for cytochrome c (green) and LC3 (red). Apoptosis was assessed by cytochrome c release and nuclear condensation/fragmentation. Autophagy was assessed by increased number of LC3 puncta. Nuclei were stained with Hoechst 33342 (blue). Bar=15 μm. Ap=apoptosis; Au=autophagy.

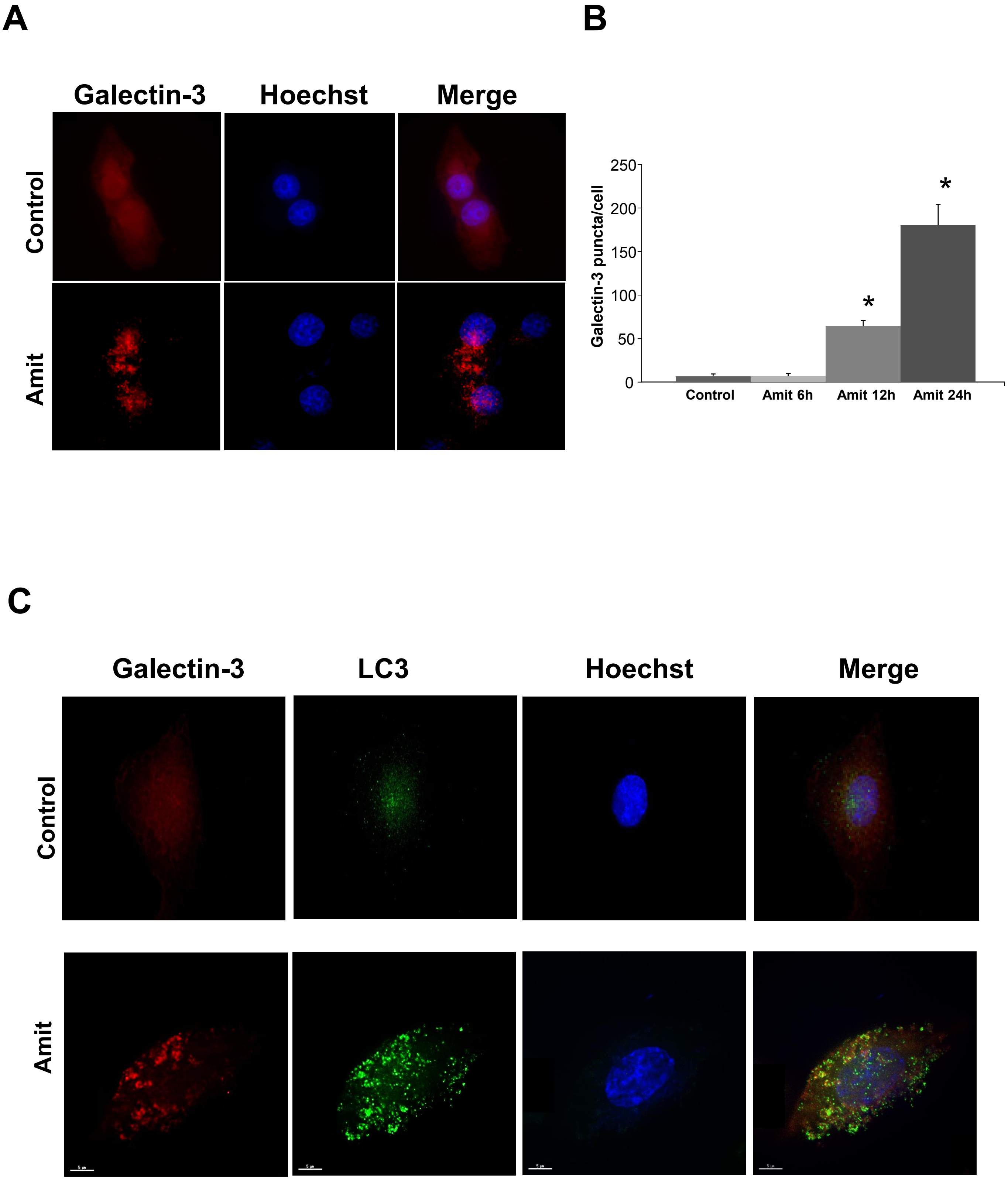

Supplementary Figure 4. (A) Representative fluorescence images of HepG2 cells that were treated with 50 μM Amitriptyline (Amit) for 12 hours. Cells were fixed and stained with anti-Galectin-3 antibodies (red) and anti-LC3 (green). Nuclei were stained with Hoechst 33342 (blue). Bar=15 μm. (**B)** Increased Galectin-3- puncta in Amit treated cells. **(C)** Colocalization of Galectin-3 and LC3 puncta. Bar=5 μm. *p<0,01, between control and Amitriptyline treated cells

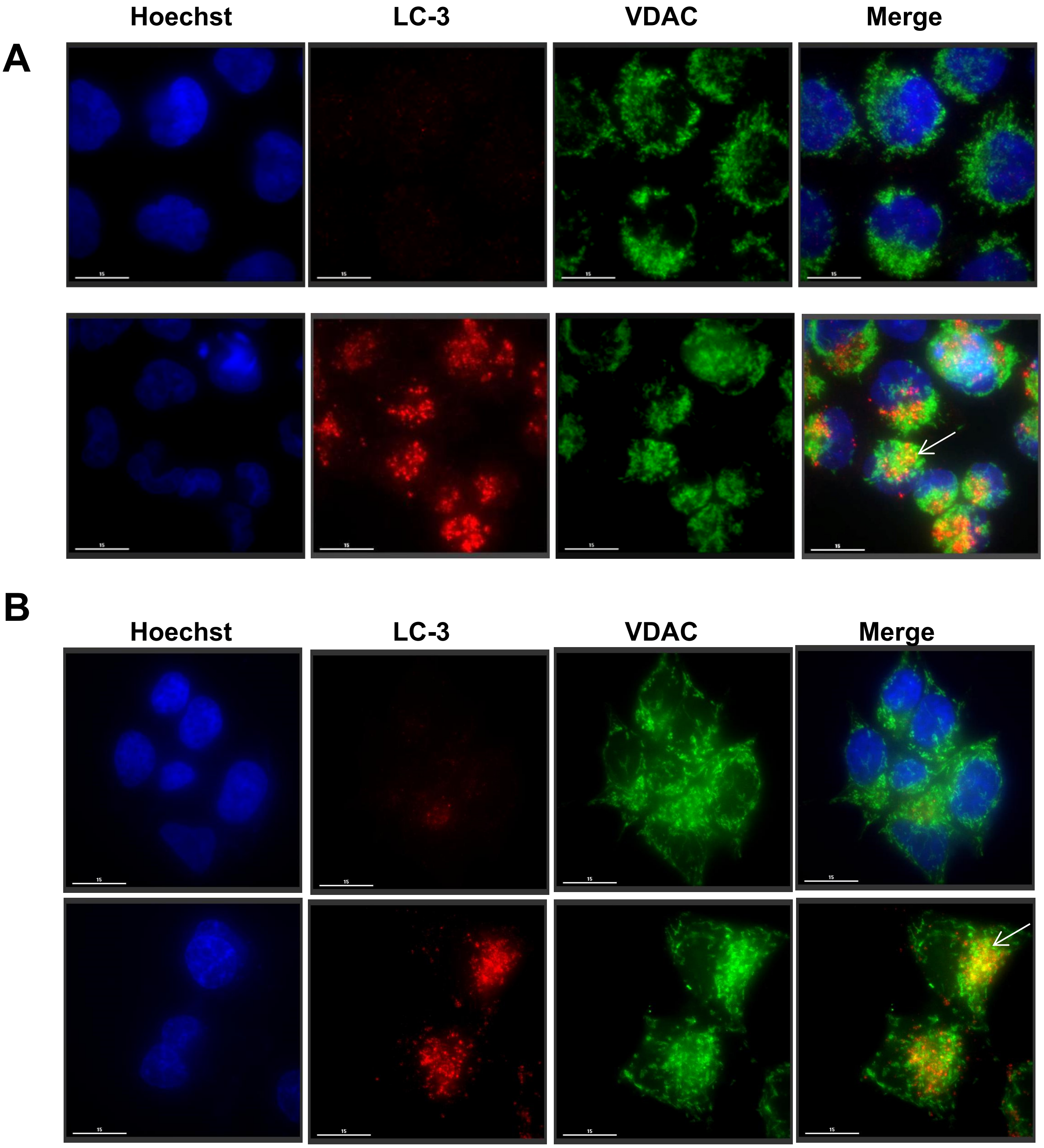

Supplementary Figure 5. Amitriptyline-induced mitophagy in H460 (A) and MCF7 (B) cells. cells were incubated in the presence or absence of 50 µM Amitriptyline for 12 h. Then, cells were fixed, and immunostained with anti-LC3 (autophagosome marker) and VDAC/Porin (mitochondrial marker) and examined in a fluorescence microscope as described in Material and Methods. Colocalization of both markers (white arrows) was assessed by the DeltaVision software. Bar = 15 µm.

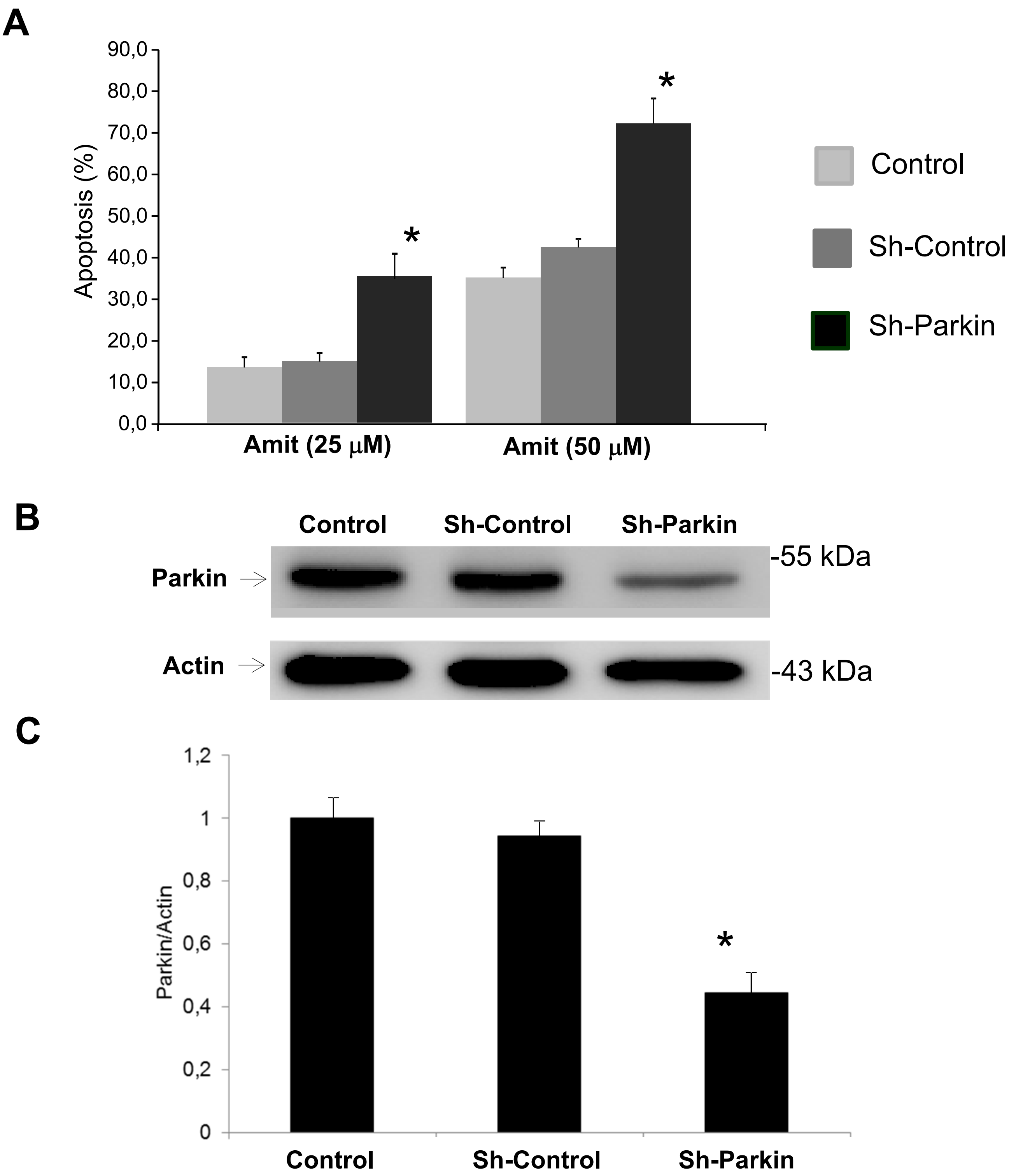

Supplementary Figure 6. (A) Down-regulation of Parkin by Parkin shRNA (h) lentiviral particles leads to early increase apoptosis under 25 or 50 μM Amitriptyline treatment for 12 hours**.** Apoptosis was analyzed by Flow cytometry as described in Material and Methods. HepG2 cells were transfected with lentiviral of a shRNA-Parkin (Sh-Parkin) construct (Santa Cruz Biotech) and a ShRNA-control (Sh-control). **(B)** The efficiency of Parkin knockdown is shown by Western blotting. Actin was used as a loading control. **(C)** Densitometry of Western blotting.* $p<0,001$.

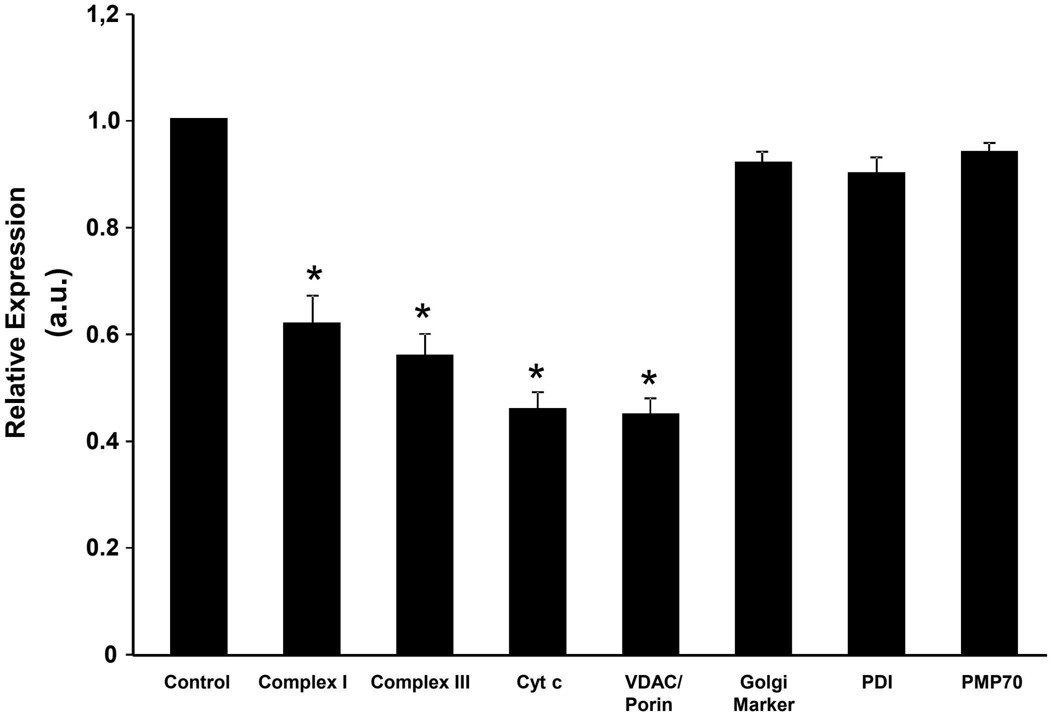

Supplementary Figure 7. Densitometry of Western blotting of Figure 5B. *p<0.01, between control and Amitriptyline treated cells. Cyt c= Cytochrome c.

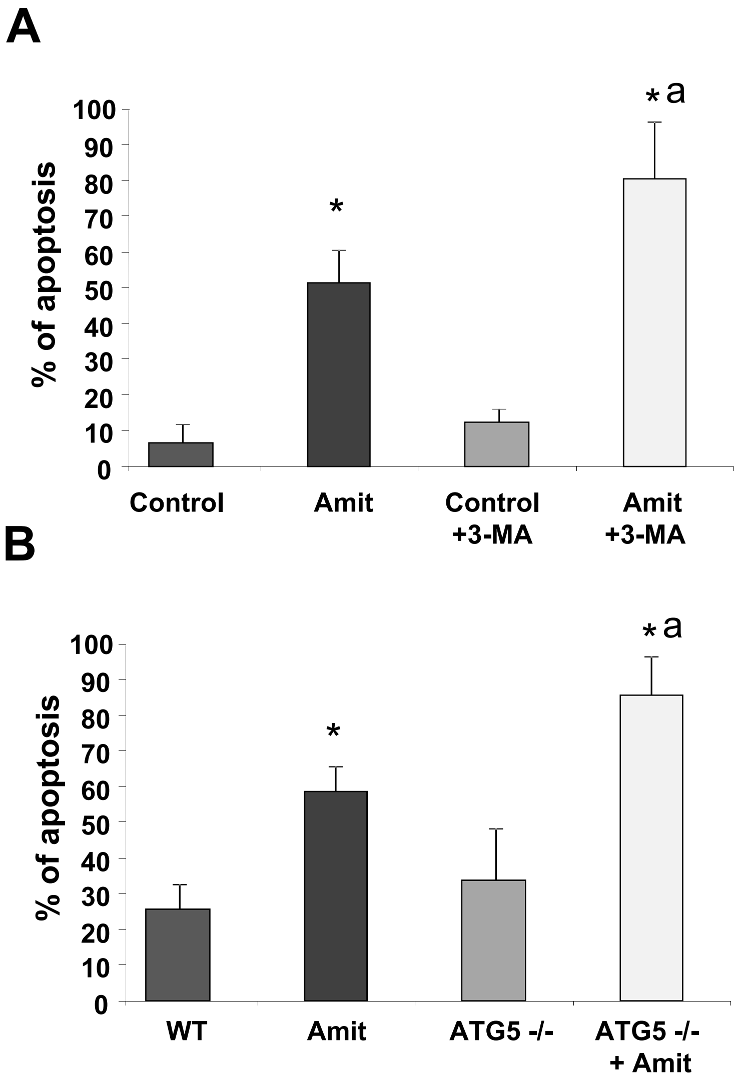

Supplementary Figure 8. Protective or pathological role of Amitriptyline in HepG2 cells. **(A)** Autophagy is initially a protective mechanism in amitriptyline-treated cells. HepG2 cells were treated for 12h with 50μM Amitriptyline (Amit) plus 20 mM 3-methyladenine (3-MA), and we determined the rate of apoptotic cells by flow cytometry as described in Material and Methods. Results are expressed as mean ± SD of three independent experiments. $^*p<0.01$ between control and Amitriptyline-treated cells. $^a p<0.01$ between the presence and absence of 3-MA. **(B)** Apoptosis is increased in Amitriptyline-treated Atg5-/- cells. Wild-type and Atg5-/- MEFs were incubated with 50 μM Amitriptyline for 24h. Apoptosis was assessed by flow cytometry in both autophagy proficient (wild-type), and autophagy-deficient cells (Atg5-/-) treated with Amitriptyline as described in Material and Methods. Results are expressed as mean ± SD of three independent experiments.$^*p < 0.01$ between the presence and the absence of Amitriptyline. $^a p < 0.01$ between Atg5-/- and wild-type MEFs.

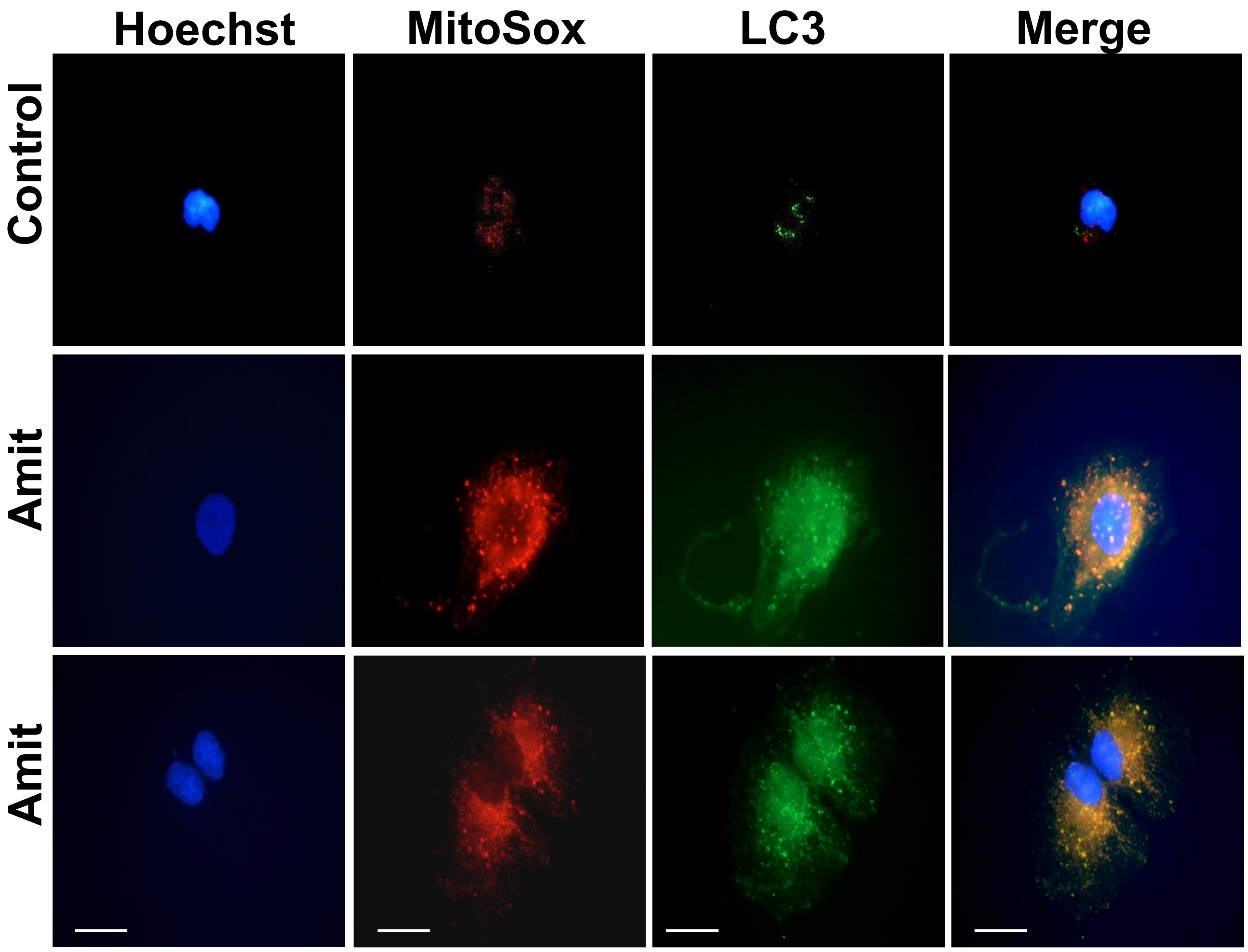

Supplementary Figure 9. ROS colocalizes with autophagosomes markers. HepG2 cells were treated with 50 μM Amitriptyline for 6 hours. Then, cells were stained with MitoSox (red), fixed and immunostained for LC3 (green Nuclei were stained with Hoechst 33342 (blue). Bar=15 μm.

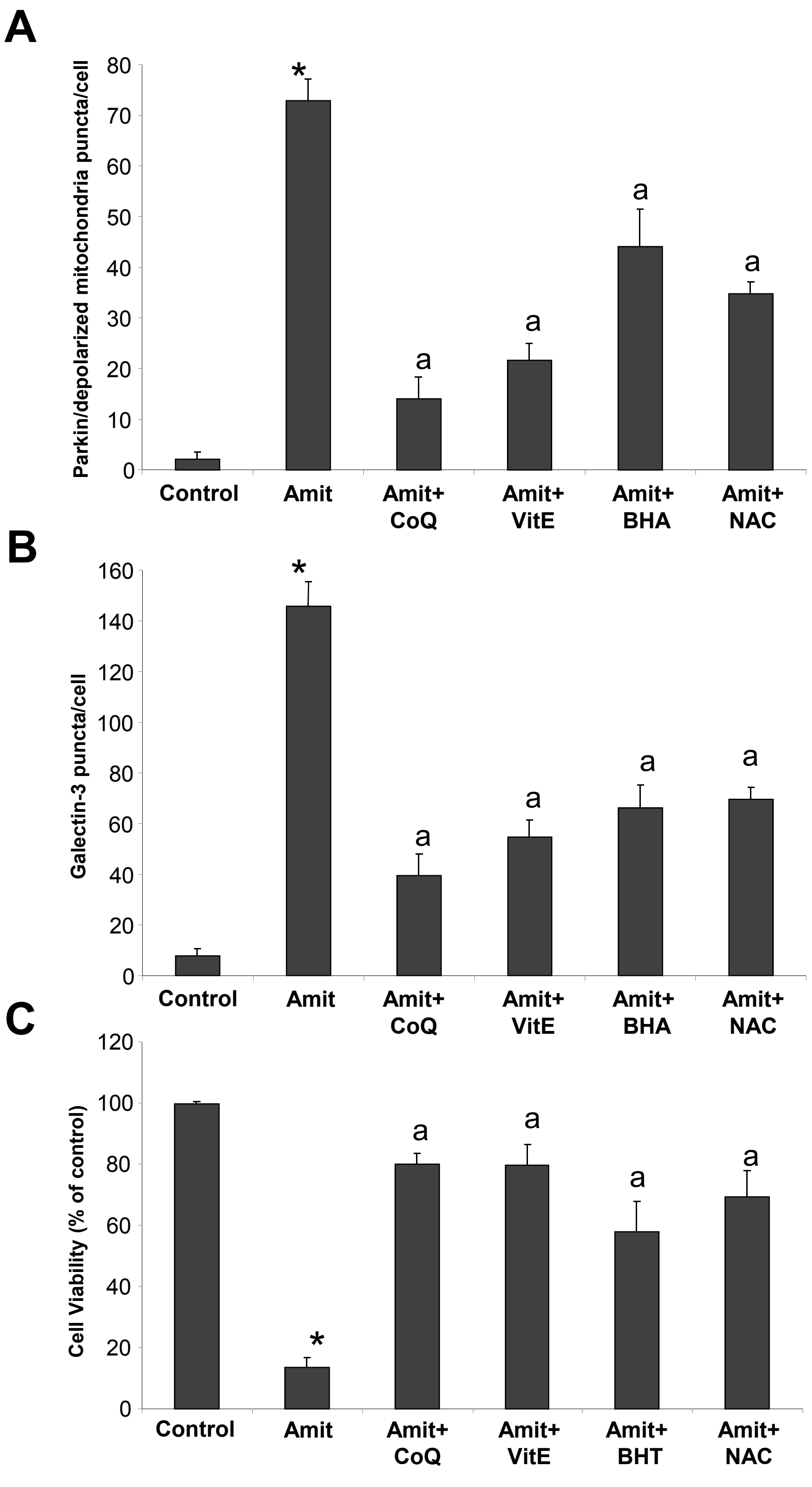

Supplementary Figure 10. Antioxidants prevent mitophagy **(A),** autophagolysosome/lysosome permeabilization **(B)** and cell death **(C)**. HepG2 cells were cultured for 24 h with 50μM Amitriptyline in the presence of CoQ. Vitamin E, BHA or NAC. Mitophagy and autophagolysosome/lysosome permeabilization were assessed by quantification of Parkin and Galectin-3 puncta per cell, respectively. Viability was analyzed by vital dye exclusion assay as described in the Materials and methods section. Data represent the mean ± SD of three separate experiments. *P<0.05, between control and Amitriptyline treated cells; ^{a}p<0.01, between the absence or presence of antioxidant.

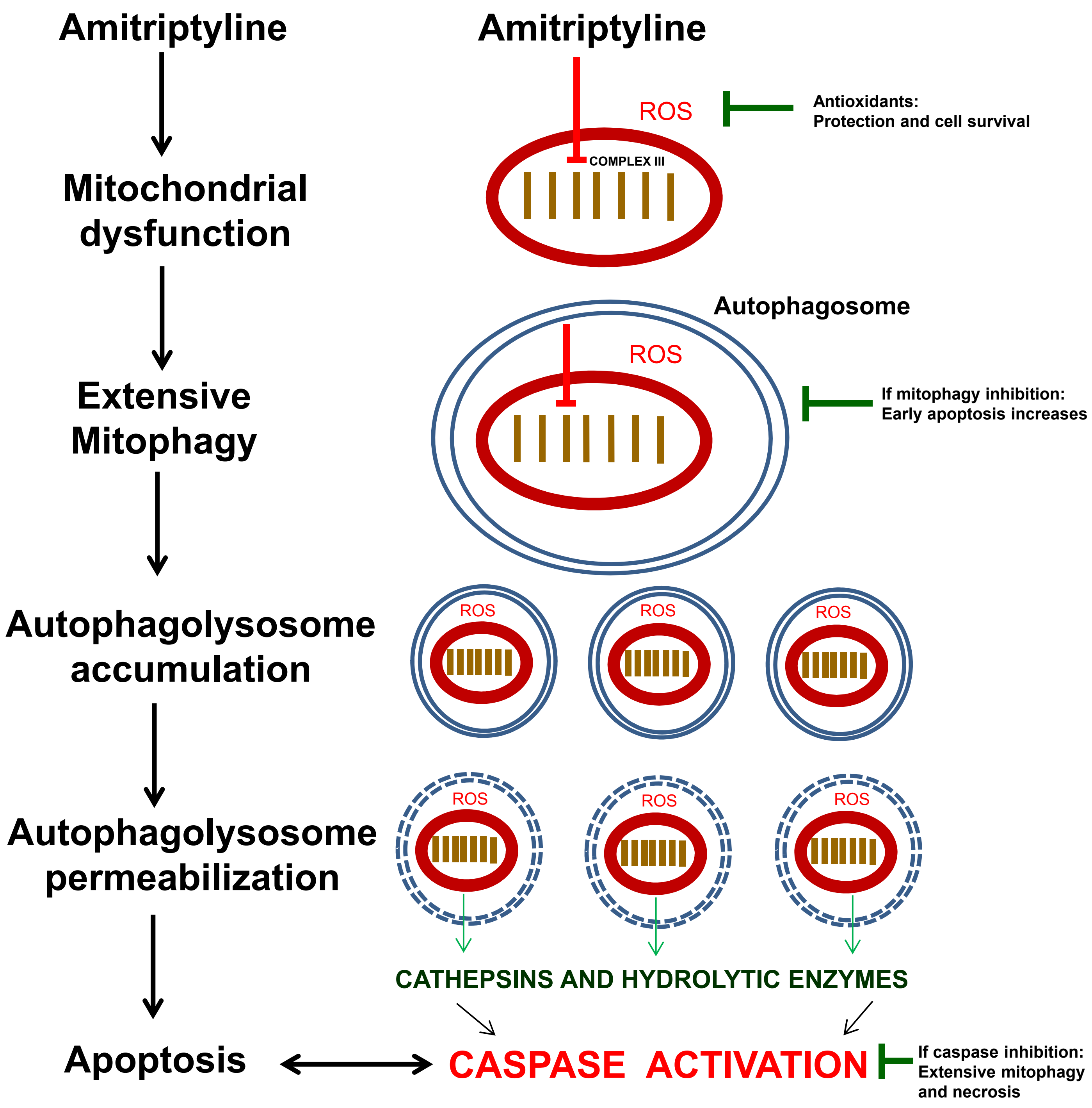

Supplementary Figure 11. Hypothetical mechanisms through which Amitriptyline induces mitophagy/apoptosis switch in HepG2 cells.
